# Efficiency Recalibrates Social‐Emotional Trade‐Offs Behind Partner Choice in Direct Reciprocity through Intention‐Specific Neural Bases

**DOI:** 10.1002/advs.202516509

**Published:** 2025-11-09

**Authors:** Rui Liao, Xintong Li, Xuqi Liu, Yu Nan, Xiaolin Zhou, Xiaoxue Gao

**Affiliations:** ^1^ Shanghai Key Laboratory of Mental Health and Psychological Crisis Intervention School of Psychology and Cognitive Science East China Normal University Shanghai 200062 China; ^2^ School of Psychological and Cognitive Sciences Peking University Beijing 100871 China

**Keywords:** affective motives, efficiency, neural bases, partner choice, reciprocity

## Abstract

Direct reciprocity, in which beneficiaries return favors to benefactors, is a cornerstone of human cooperation. Previous empirical work addresses partner control—how individuals decide whether and how much to reciprocate—whereas the equally critical dimension of partner choice, deciding whom to reciprocate when aided by multiple benefactors, remains understudied. This gap is addressed by testing two determinants: social‐emotional motivations and reciprocal efficiency (the efficiency of reinforcing future relational capital). Combining an interpersonal task with fMRI multivariate neural expressions and representational similarity analysis, it is demonstrated that efficiency recalibrates choices between altruistic and strategic benefactors by shifting the neural balance between distinct social‐emotional concerns. When reciprocating to altruistic benefactors yielded higher efficiency, participants prioritized communal concerns (gratitude/guilt) represented in the ventromedial prefrontal cortex, whereas higher efficiency for strategic benefactors led them to prioritize obligation represented in the ventral striatum. General efficiency engaged the putamen, dorsomedial prefrontal, and orbitofrontal cortices; the inferior parietal lobe integrated efficiency‐driven recalibration. These findings suggest that efficiency does not merely optimize material outcomes but adaptively reweights social‐emotional concerns behind reciprocal partner choices, bridging economic models of rational choice with psychological theories of social emotions, offering insights into human cooperation and related practical applications.

## Graphical abstract

Bridging the gap between two crucial mechanisms of cooperation, namely direct reciprocity and partner choice, this study reveals the neurocognitive bases underlying how individuals prioritize reciprocal partner when receiving help from multiple benefactors with diverse intentions and reciprocal efficiency. The efficiency‐driven reciprocal decisions are not purely rational but balance intention‐specific neurocognitive trade‐offs between social‐emotional motives of communal concern and obligation.

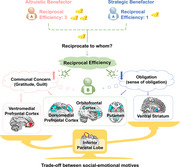

## 1 Introduction

Cooperation is crucial for human survival, adaptation, and development.^[^ ^1^, ^2^, ^3^, ^4^ ^]^ Among the several mechanisms proposed for the evolution and formation of cooperation, direct reciprocity has been one of the most extensively studied across disciplines such as psychology,^[^ ^3^, ^5^, ^6^ ^]^ cognitive neuroscience,^[^ ^7^, ^8^, ^9^, ^10^ ^]^ economics,^[^ ^6^, ^11^, ^12^, ^13^ ^]^ and evolutionary biology.^[^ ^3^, ^4^, ^14^, ^15^, ^16^ ^]^ The theory of direct reciprocity posits that, the beneficiary receives a favor from the benefactor and responds with reciprocal behavior, thereby enabling the generation of self‐sustaining non‐kin cooperative relationships through pairwise interactions.^[^ ^17^, ^18^, ^19^ ^]^ Based on this theoretical foundation, prior cognitive neuroscience research on direct reciprocity has predominantly employed paradigms grounded in the partner control perspective.^[^ ^7^, ^12^, ^20^, ^21^, ^22^, ^23^, ^24^, ^25^, ^26^ ^]^ These paradigms typically investigate the cognitive and neural mechanisms of how individuals decide whether and to what extent to reciprocate in order to incentivize future cooperation or punish non‐cooperation, within pairwise interactions with one partner or one of multiple partners.^[^ ^20^, ^21^, ^23^, ^24^, ^26^ ^]^ However, this partner control‐centric approach has largely overlooked another significant scientific question: When receiving help from multiple benefactors, how does the beneficiary decide whom to prioritize for reciprocity (i.e., partner choice)?

Both real‐world scenarios and established cooperation theories underscore the prevalence and significance of partner choice within the contexts of direct reciprocity. In daily life, individuals facing serious economic pressure due to illness, businesses encounter cash flow shortages, or countries confront major disasters, they may receive help from multiple sources. However, constrained by the resource limitations inherent in the help‐seeking state,^[^ ^27^, ^28^ ^]^ they can typically only choose one benefactor for prioritized reciprocation and relationship reinforcement. Theoretically, previous research has proposed that partner choice is another important mechanism for the evolution of cooperation, independent of direct reciprocity.^[^ ^14^, ^29^, ^30^ ^]^ This mechanism conceptualizes individuals as existing within a biological market of potential partners, where they can therefore choose or reject social partners.^[^ ^31^, ^32^, ^33^, ^34^, ^35^, ^36^ ^]^ It fosters competitive altruism, wherein multiple altruists compete to provide benefits in order to capturing the beneficiary's attention and being selected as a cooperation partner to secure future cooperative gains.^[^ ^31^, ^37^, ^38^, ^39^, ^40^, ^41^ ^]^ This theoretically confirms the possibility and importance of the beneficiary receiving help from multiple sources and subsequently selecting whom to reciprocate. What are the cognitive and neural mechanisms underlying the beneficiary's partner choice during reciprocity? Given that partner choice and direct reciprocity are typically studied as separate mechanisms, especially in the fields of cognitive neuroscience,^[^ ^7^, ^8^, ^9^, ^10^, ^42^, ^43^, ^44^ ^]^ this question remains unexplored in cognitive science despite the fact that it lies at the heart of understanding both reciprocity‐based and partner choice‐based cooperation. Here, we address this gap by concentrating on two potential important determinants underlying the partner choice in direct reciprocity: 1) the emotional motivational trade‐off elicited by perceived benefactor's intentions, and 2) the efficiency of reinforcing future relational capital.

On one hand, previous research suggests that the perception of partner's intention serves as a common cognitive mechanism for both partner control based direct reciprocity and partner choice. While not directly situated within the context of favor‐receiving and reciprocity, research has demonstrated that the perceived intention of the partner is a significant factor influencing partner choice,^[^ ^30^, ^34^, ^45^ ^]^ and its impact on partner choice is even more pronounced than in partner control studies.^[^ ^30^, ^34^, ^46^ ^]^ Research on direct reciprocity in pairwise interactions has shown that, beneficiaries make trade‐off between two types of social‐emotional motivations derived from benefactors’ different intentions in order to decide the amount of reciprocity.^[^ ^47^, ^48^, ^49^, ^50^ ^]^ Some benefactors are altruistic, offering genuine support without expecting anything in return. This altruistic behavior primarily evokes feelings of gratitude and guilt in the beneficiary, thereby fostering a communal motivation for reciprocity.^[^ ^19^, ^47^, ^50^, ^51^, ^52^, ^53^, ^54^, ^55^, ^56^, ^57^, ^58^ ^]^ Other benefactors may be strategic, providing help with the expectation of reciprocation. This strategic behavior predominantly elicits a sense of obligation in the beneficiary, driven by the norm of reciprocity, thus promoting reciprocity through an obligation motivation.^[^ ^47^, ^48^, ^49^, ^50^, ^52^, ^59^, ^60^, ^61^, ^62^, ^63^, ^64^, ^65^ ^]^ These distinct motivations are supported by dissociable neural systems: communal motivation primarily engages the ventromedial prefrontal cortex (vmPFC) and anterior insula (aINS), whereas obligation motivation mainly recruits the dorsomedial prefrontal cortex (dmPFC) and temporoparietal junction (TPJ).^[^ ^47^, ^51^, ^52^, ^66^ ^]^ The final reciprocal decision, which relies on the integration and weighting of these motives, involves regions such as the inferior parietal lobe (IPL) and dorsolateral prefrontal cortex (dlPFC).^[^ ^47^, ^67^, ^68^ ^]^ In this view, when confronted with multiple benefactors with divergent intentions, beneficiaries should be compelled to navigate the resultant social‐emotional dilemma in order to make a partner choice, given that both types of motivations—communal and obligation—serve to promote the act of reciprocity. At the individual level, these reciprocal decisions are critical for maintaining social relationships and ensuring survival adaptation, particularly under resource‐scarce conditions.^[^ ^19^, ^54^, ^69^, ^70^, ^71^ ^]^ At the societal level, these decisions crucially influence the flow of social resources, determining the extent to which resources flow toward altruistic versus strategic cooperators,^[^ ^33^, ^34^, ^38^, ^72^ ^]^ ultimately shaping distinct types of social relational networks.^[^ ^73^, ^74^, ^75^, ^76^, ^77^ ^]^ Therefore, it is important to study the mechanisms underlying partner choice in reciprocity from the perspective of the social emotional motivational trade‐off elicited by perceived benefactor's intentions.

On the other hand, reciprocity and partner choice share a common adaptive goal: optimizing future cooperative relational capital.^[^ ^3^, ^30^, ^78^, ^79^, ^80^, ^81^ ^]^ In everyday scenarios, given the variations in benefactor characteristics—such as differences in resource endowment and the valuation placed on reciprocation from others—a unit of reciprocated resource may exert a potent effect on relationship maintenance for some benefactors, while having a minimal impact on others.^[^ ^6^, ^32^, ^57^, ^82^, ^83^ ^]^ Thus, the consideration of reciprocal efficiency to the benefactor—the capacity to maximize relational gains per unit of reciprocated resource—may be a key foundation for the partner choice of direct reciprocity. In fact, operationalized as the magnitude of collective gains (e.g., total benefits for both parties/groups in resource allocation tasks) or the exchange ratio of output per unit of input (e.g., proportion of collective financial growth per unit investment in trust games or public goods games), prior work demonstrates that efficiency preference critically drives cooperative behaviors, such as resource allocation in dictator or ultimatum games,^[^ ^84^, ^85^, ^86^, ^87^ ^]^ investment in trust games,^[^ ^88^, ^89^, ^90^ ^]^ altruistic acts in donation tasks,^[^ ^68^, ^91^, ^92^, ^93^ ^]^ and individual contributions to group welfare in public good games.^[^ ^94^, ^95^, ^96^ ^]^ Moreover, a series of studies have dissociated efficiency from other important motivations underlying resource allocation, i.e., greed, and inequity aversion, revealed how individuals make decisions when faced with tradeoff or conflicts between these three motivations,^[^ ^97^, ^98^, ^99^, ^100^, ^101^, ^102^, ^103^ ^]^ and link efficiency processing to the activities in the striatum^[^ ^98^ ^]^ and medial orbitofrontal cortex (mOFC).^[^ ^104^ ^]^

How does reciprocal efficiency shape preferences for altruistic versus strategic benefactors? Does reciprocal efficiency recalibrate the balance between feelings of communal concern and obligation behind reciprocity, or simply contribute as an independent economic concern? Whether efficiency recalibrates social‐emotional trade‐offs behind reciprocal partner choice through benefactor‐intention‐specific neural bases? Answering these research questions is critical for a mechanistic understanding of partner choice in direct reciprocity, yet they remain unaddressed by prior research on efficiency in cooperation and studies on partner‐control‐based reciprocity for two reasons. For one thing, although existing work has examined how efficiency modulates proactive cooperative behaviors, such as resource allocation, trust‐based investment, charity, or public good contributions,^[^ ^2^, ^105^, ^106^, ^107^ ^]^ reciprocity, which rooted in the beneficiary's responsively evaluation of and reaction to a benefactor's prior action, likely engages cognitive processes that differ from those underlying proactive cooperation. For example, the trade‐off between communal and obligation motivations is unique to reciprocal contexts and absent in proactive cooperation.^[^ ^47^, ^52^, ^56^, ^59^, ^60^, ^66^, ^108^ ^]^ For another, while extensive research has delineated the cognitive and neural mechanisms of partner‐control‐based reciprocity in pairwise interactions, emerging evidence suggests potential psychological distinctions between partner choice and partner control decision‐making, such as the increased involvement of theory‐of‐mind and intention inferences in partner choice.^[^ ^7^, ^8^, ^9^, ^10^, ^34^, ^109^ ^]^ The distinctive psychological features of reciprocal partner choice necessitate the development of dedicated experimental paradigms to investigate its mechanisms.

**Figure 1:**
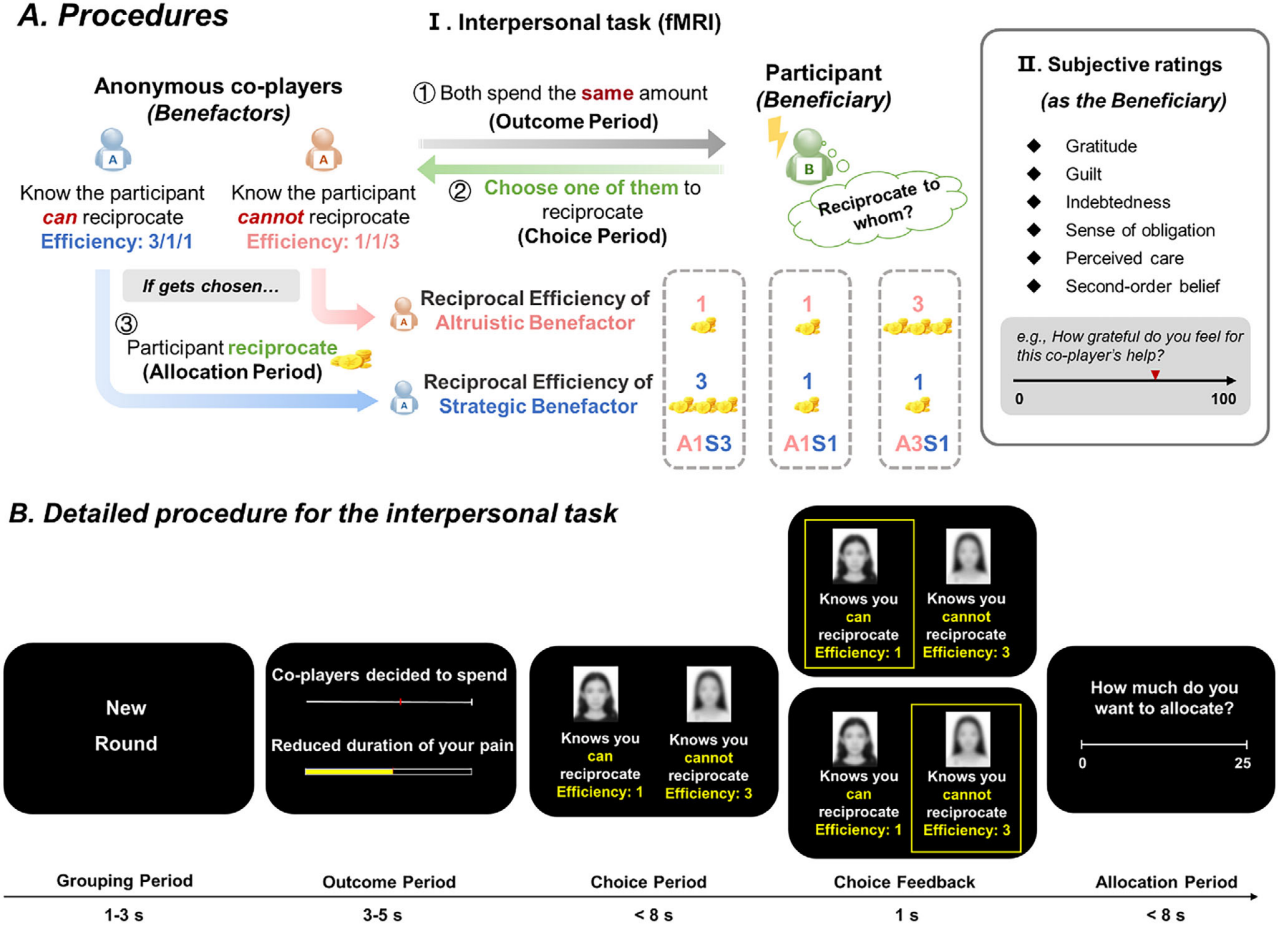
Experimental procedure. A) General procedures. In the interpersonal task (I), participants would receive two 20 s pain stimulations per trial and interacted with two anonymous benefactors, each of whom had decided whether, and how much, to spend from their endowment to reduce the duration of the pain stimulation the participants were assigned to, with each benefactor corresponding to one of the two stimulations. One benefactor (i.e., Strategic Benefactor) was aware that participants could reciprocate, while the other (i.e., Altruistic Benefactor) was not. Despite this, both benefactors spent the same amount of endowment to help. Endowed with 25 yuan, participants were required to select one benefactor to reciprocate to (i.e., reciprocal partner choice) and decide how much to allocate to this chosen benefactor (i.e., monetary allocation), considering varying reciprocal efficiencies (1 or 3). Three distinct conditions were established: 1) A1S3, where the Altruistic Benefactor had an efficiency of 1 and the Strategic Benefactor had an efficiency of 3; 2) A1S1, where both benefactors had an efficiency of 1; and 3) A3S1, where the Altruistic Benefactor had an efficiency of 3 and the Strategic Benefactor had an efficiency of 1. With reciprocal efficiency being 1, for every 1 yuan the participant reciprocated, the benefactor would receive 1 yuan; with the reciprocal efficiency being 3, for every 1 yuan the participant reciprocated, the benefactor would receive 3 yuan. To note, participants were instructed that all the benefactors made their decisions without knowledge of the reciprocal efficiency. After the interpersonal task, participants recalled how much they believed the benefactor cared about them (i.e., perceived care), and how much the benefactor expected for repayment (i.e., second‐order belief), as well as their feelings of gratitude, guilt, obligation, and indebtedness in response to each benefactor's help (II. Subjective ratings). B) Detailed procedure for the interpersonal task. Each trial began by informing the participant that the program had randomly chosen two benefactors for the current trial (Grouping Period, 1–3 s). Then, information regarding the two benefactors’ spending and the corresponding amount of pain reduction for the participant was presented (Outcome Period, 3–5 s). Subsequently, participants would see the two benefactors’ blurred photos and IDs, presented on the left and right sides of the screen, respectively. The information that the benefactor knew the participant could or could not repay, as well as the reciprocal efficiency of each benefactor, were given underneath the corresponding photo. The participant had to choose one benefactor to reciprocate (i.e., reciprocal partner choice) by pressing the left or the right button (Choice Period, < 8 s). A box will appear around the selected benefactor and last for 1 s (Choice Feedback, 1 s). At the end of each trial, the participant was endowed with 25 yuan and decided how much to allocate to the chosen benefactor as reciprocity (Allocation Period, < 8 s, continuous choice from 0 to 25, step of 1 yuan). Unbeknownst to the participants, all decisions of the benefactors were predetermined by the computer program.

To bridge this gap and address the aforementioned research questions, first, we developed an interpersonal task (**Figure** **1**) to simulate the dilemma of reciprocal partner choice, based on our well‐established interpersonal task originally designed to study partner‐control‐based reciprocity (i.e., deciding how much to reciprocate in pairwise interactions)^[^ ^47^ ^]^ (see Experimental Section). During functional magnetic resonance imaging (fMRI) scanning, in each trial, participants receive equivalent favors from two anonymous benefactors but could only choose one of them to reciprocate. We manipulated the intentions of the two benefactors by providing extra information: when deciding whether to help the participant, one benefactor knew the participant could not repay after receiving help given the experimental setting (Altruistic Benefactor), while the other knew the participant could decide how to repay after receiving help (Strategic Benefactor). Moreover, the efficiency of reciprocating to the two benefactors might vary, being 1 or 3 as determined randomly by the computer program. With reciprocal efficiency at 1, the benefactor receives 1 yuan per yuan reciprocated; with reciprocal efficiency at 3, the benefactor receives 3 yuan per yuan reciprocated. This formed three reciprocal conditions: higher reciprocal efficiency of the Strategic Benefactor (A1S3), equal reciprocal efficiency of the two benefactors (A1S1), and higher reciprocal efficiency of the Altruistic Benefactor (A3S1). Under this manipulation of efficiency, higher reciprocal efficiency signifies a greater capacity to reinforce future cooperative relational capital per unit resource reciprocated, aligning with the adaptive goals of direct reciprocity and partner choice.^[^ ^3^, ^30^, ^78^, ^79^, ^80^, ^81^ ^]^ Moreover, to increase the variability of the task, the cost of the two benefactors was parametrically manipulated (9 levels: 4, 6, 8, 10, 12, 14, 16, 18, and 20). As manipulation checks, after fMRI scanning, the participants rated their feelings of communal concern (i.e., gratitude and guilt) and obligation, as well as the perceived altruistic and strategic intentions, when receiving help from each benefactor.

Second, we examine whether and how reciprocal efficiency shape preferences for altruistic versus strategic benefactors by recalibrating the balance between the two emotional motivations of common concern and obligation. Behaviorally, using emotional ratings, we calculated and compared the relative contributions of these two emotional motivations to the reciprocal partner choices under different reciprocal efficiency conditions. We also conducted analysis on the amounts of reciprocity to the chosen benefactors to test whether participants’ relative concerns for self‐interest vary with reciprocal efficiency. Neurally, we conducted cross‐study neural expressions for the processing of communal and obligation motivations using the whole‐brain multivariate pattern maps established by our recent research on how these two motivations contribute to partner‐control‐based reciprocity (an independent fMRI dataset).^[^ ^47^ ^]^ We computed the neural relative weight of the two emotional motivations and examined their variations across different efficiency conditions. To rule out that reciprocal efficiency altered the intensity rather than the relative weighting of the two emotional motivations, a possibility that may not be captured by post‐scan ratings, we conducted an additional behavioral experiment (see *Supplementary Methods*). The procedure was identical to the fMRI experiment, except that during the interpersonal task, participants reported their emotional feelings and appraisals after the efficiency information was presented, but prior to finalizing their reciprocal decision.

Third, we employed representational similarity analysis (RSA) on fMRI data to identify the specific brain regions supporting the recalibration effect of efficiency on the social‐emotional trade‐offs behind reciprocal partner choice, and whether these processes involve benefactor‐intention‐specific neural representations. Four cognitive representational dissimilarity matrices (RDMs) were built to identify brain regions that characterized four different cognitive processes: the processing related to the effect of efficiency on reciprocal partner choices, the processing of Altruistic Benefactor's efficiency, the processing of Strategic Benefactor's efficiency, and the general processing of reciprocal efficiency.

## 2 Results

### 2.1 Reciprocal Efficiency Influenced Participants’ Reciprocal Partner Choices

**Figure 2:**
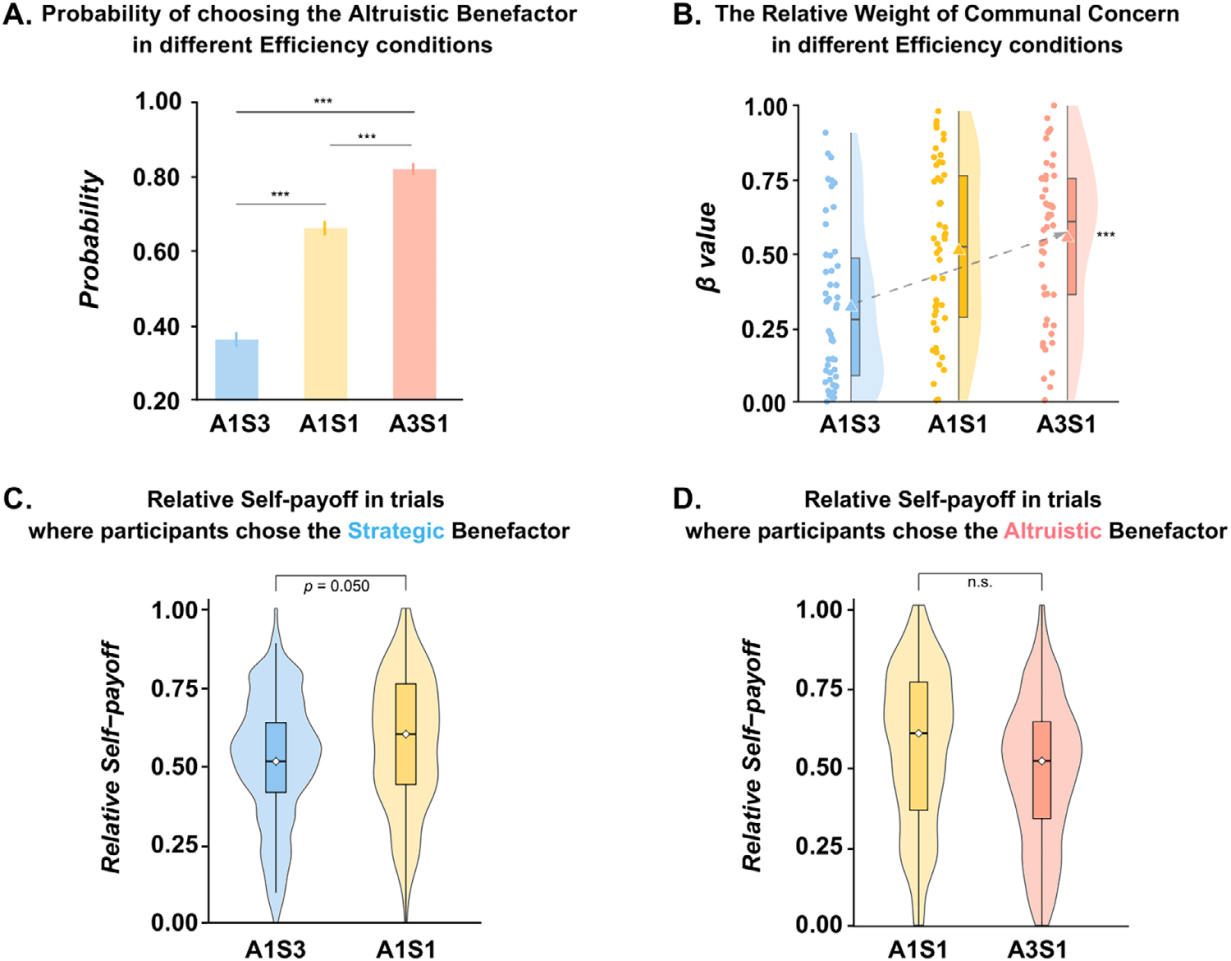
Behavioral results. A) The probability of choosing to reciprocate to Altruistic Benefactor in three reciprocal efficiency conditions. Linear trend analyses revealed that the probability of choosing Altruistic Benefactors increased linearly from the A1S3 condition, A1S1 condition to A3S1 condition. Data are presented as mean values ± SEM (*n* = 50). B) The relative weight of Communal Concern in three reciprocal efficiency conditions. Results revealed a significant linear increase of the relative weight of Communal Concern (defined as $\frac{|\beta communal\;factor|\;}{(\left|\beta communal\;factor|+|\beta obligation\;factor\right|)\;}$) with the increasing reciprocal efficiency of the Altruistic Benefactor (i.e., from A1S3 to A1S1 and A3S1). Each dot represents the average relative weight of Communal Concern in three reciprocal efficiency conditions for each participant (*n* = 50). C) The Relative Self‐payoff in trials where participants chose to reciprocate to the Strategic Benefactor. We defined the “Relative Self‐payoff” as ‘$\frac{the\;amount\;reserved\;for\;oneself}{\left(the\;amount\;reserved\;for\;oneself+efficiency\;*\;the\;allocation\;to\;the\;benefactor\right)}$’, representing the relative concern for self‐interest in relative to the concern for the chosen benefactor's interest. We compared the Relative Self‐payoff when participants chose the Strategic Benefactor in the A1S3 and A1S1 conditions using the Mann‐Whitney U Test (see *Methods*), which demonstrated how the increased efficiency of the Strategic Benefactor modulated the Relative Self‐payoff. Results showed that the Relative Self‐payoff decreased as the efficiency of reciprocating the Strategic Benefactor increased. D) The Relative Self‐payoff in trials where participants chose to reciprocate to the Altruistic Benefactor. We conducted similar analysis on the Relative Self‐payoff when participants chose the Altruistic Benefactor, and no significant difference in the Relative Self‐payoff was observed between the A3S1 and A1S1 conditions. Data are shown as violin plots depicting the distribution of individual data points (total *n* = 50). The white diamond and black bar indicate the mean and the interquartile range, respectively. Statistical comparisons between conditions were performed using two‐tailed Mann–Whitney U tests. Data are presented as raw values normalized within participants. p values are indicated above the plots. Significance: *n.s*. indicates a non‐significant effect; *** *p* < 0.001.

A 3 (Efficiency: A1S3, A1S1, and A3S1) × 9 (Benefactor's Cost: 4, 6, 8, 10, 12, 14, 16, 18, and 20) repeated‐measures analysis of variance (ANOVA) revealed significant main effects of Efficiency (Greenhouse‐Geisser correction: *F*_(1.64, 80.55)_ = 50.065, *p* < 0.001, *η_p_^2^* = 0.505) and Benefactor's Cost (Greenhouse‐Geisser correction: *F*_(3.02, 147.89)_ = 3.372, *p* = 0.020, *η_p_^2^* = 0.064) on the probability of choosing Altruistic Benefactor (**Figure** **2**A). The interaction effect between these two variables was not significant (Greenhouse‐Geisser correction: *F*_(7.94, 388.92)_ = 0.989, *p* = 0.444, *η_p_^2^* = 0.020). Specifically, the probability of choosing Altruistic Benefactors was significantly higher in A3S1 condition than in A1S3 condition (*mean difference* = 0.461, *Standard Error* = 0.056, *95%CI* = [0.349, 0.574], *p* < 0.001) and A1S1 condition (*mean difference* = 0.158, *Standard Error* = 0.037, *95%CI* = [0.084, 0.232], *p* < 0.001). Linear trend analyses showed that, the probability of choosing Altruistic Benefactors increased linearly from the A1S3 condition, A1S1 condition to A3S1 condition (*F*_(1,49)_ = 68.309, *p* < 0.001, *η_p_^2^* = 0.582). These results were replicated in an additional behavioral experiment (see *Supplementary Results*). Note, since we did not observe a significant interaction effect between Efficiency and Benefactor's Cost, and we primarily focused on the effect of Efficiency in the current study, we combined the data of all levels of Benefactor's Cost in the subsequent analyses.

### 2.2 Reciprocal Efficiency Modulated the Trade‐Off Between the Feelings of Communal Concern and Obligation Underlying Reciprocal Partner Choices

Next, we aimed to explore the roles of feelings of communal concern (gratitude and guilt) and obligation (sense of obligation) underlying the effect of Efficiency on reciprocal partner choices. As stated above, the current interpersonal task was adapted from the recent study,^[^ ^47^ ^]^ which adopted a perspective of partner control, and aimed to reveal how beneficiaries generate, integrate and weigh these two emotional motivations in order to make decisions of how much to reciprocate to a specific benefactor with altruistic or strategic intention (see Experimental Section). Although the present study and this previous research^[^ ^47^ ^]^ differ fundamentally in the research question and the type of reciprocal choice participants make, they share a similar experimental foundation, i.e., both involving the generation and weighting of beneficiary's emotions of gratitude, guilt and obligation after receiving help. Therefore, as manipulation checks of the inductions and measurements of emotions and appraisals, we conducted two lines of analyses on participants’ subjective ratings referring to this previous research.^[^ ^47^ ^]^ First, the results of paired‐sample *t*‐tests replicated previous research,^[^ ^47^ ^]^ showing that participants perceived more care from help (*mean difference* = 19.136, *Standard Error* = 1.374, *95%CI* = [16.376, 21.896], *t_(49)_* = 13.931, *p* < 0.001) and reported significantly more gratitude (*mean difference* = 18.896, *Standard Error* = 1.329, *95%CI* = [16.224, 21.568], *t_(49)_* = 14.213, *p* < 0.001), guilt (*mean difference* = 14.568, *Standard Error* = 2.367, *95%CI* = [9.811, 19.325], *t_(49)_* = 6.154, *p* < 0.001) to the Altruistic Benefactor than to the Strategic Benefactor (Figure S1A, Supporting Information). Meanwhile, participants believed that the benefactors expected them to reciprocate more (*mean difference* = 48.432, *Standard Error* = 3.521, *95%CI* = [41.357, 55.507], *t_(49)_* = 13.756, *p* < 0.001), and reported significantly more sense of obligation (*mean difference* = 11.960, *Standard Error* = 2.712, *95%CI* = [6.510, 17.410], *t_(49)_* = 4.410, *p* < 0.001) to the Strategic Benefactor than to Altruistic the Benefactor. In line with previous research^[^ ^47^ ^]^ these effects were magnified as the benefactor's cost increased (Figure S1B, Supporting Information).

Second, to reduce the dimensionality of data and extract the principal components of communal concern and obligation from these appraisals and emotions, we conducted Confirmatory Factor Analysis (CFA) on these subjective ratings of appraisals and emotions referring to the previous research.^[^ ^47^ ^]^ This analysis extracted two principal factors, namely the communal factor and obligation factor, which could explain 75% of the variance in participants’ subjective ratings. The communal factor reflected participants’ perception that the benefactor cared about their welfare and resulted in emotions of guilt and gratitude, while the obligation factor reflected participants’ second‐order beliefs about the benefactor's expectation for repayment and the sense of obligation.

Then, we tested whether the contributions of these two emotional motives to reciprocal choices varied across different Efficiency condition, we extracted the regression coefficients (*βs*) of communal and obligation factors for predicting the probability of choosing the Altruistic Benefactor in each of the three Efficiency conditions of each participant and defined “the relative weight of Communal Concern” as ‘$\frac{|\beta communal\;factor|\;}{(\left|\beta communal\;factor|+|\beta obligation\;factor\right|)\;}$’ (see Experimental Section). A one‐way (Efficiency: A1S3, A1S1, A3S1) repeated‐measures analysis of variance (ANOVA) was conducted on the relative weight of Communal Concern. Results revealed a significant main effect of Efficiency (Greenhouse‐Geisser correction: *F*_(1.69, 82.80)_ = 11.003, *p* < 0.001, *η_p_^2^* = 0.183; Figure 2B). Direct comparison revealed a significant linear increase of the relative weight of Communal Concern with the increasing reciprocal efficiency of the Altruistic Benefactor (i.e., from A1S3 to A1S1 and A3S1; *F*_(1,49)_ = 21.714, *p* < 0.001, *η_p_^2^* = 0.307). Specifically, the relative weight of Communal Concern in the A3S1 condition was significantly higher compared with the A1S3 condition (*mean difference* = 0.285, *Standard Error* = 0.061, *95%CI* = [0.162, 0.408], *p* < 0.001) and was marginally significantly higher compared with the A1S1 condition (*mean difference* = 0.123, *Standard Error* = 0.071, *95%CI* = [‐0.020, 0.266], *p* = 0.091). Analysis on the absolute weights for Communal Concern and Obligation yielded a similar pattern of results (see *Supplementary Results* and Figure S6, Supporting Information). These results indicated that reciprocal efficiency may modulate the trade‐off between feelings of communal concern and obligation underlying the reciprocal partner choices.

In the fMRI experiment, participants made subjective ratings of emotions after the interpersonal task, without knowing the information of reciprocal efficiency. This setting was not able to exclude the possibility that reciprocal efficiency may modulate the intensities of emotions toward the benefactors, in addition to the trade‐off between the two emotional motives. Therefore, we conducted an additional behavioral experiment (see *Supplementary Methods*), the procedure of which was identical to the interpersonal task of the fMRI experiment, except that during the interpersonal task, participants were required to evaluate their emotional feelings and appraisals after knowing the reciprocal efficiency of each benefactor and before deciding whom to reciprocate. Results remained the same as previous research^[^ ^47^ ^]^ and the results of our fMRI experiment (Figure S1, Supporting Information). More importantly, the Efficiency did not significantly influence participants’ subjective ratings of appraisals and emotions, and did not significantly interact with the Benefactor Type or Benefactor's Cost (Figure S2 and Table S1, Supporting Information). These findings, taken together, revealed that reciprocal efficiency influenced participants' reciprocal partner choices by modulating the trade‐off between feelings of communal concern and obligation while making decisions, rather than the intensity of their emotional feelings.

### 2.3 The Relative Concern for Self‐Interest did not Increase with Reciprocal Efficiency

While we could not estimate participants’ concerns for self‐interest directly from their reciprocal partner choices given the current experimental design, we were able to infer these preferences from the participants’ amounts of allocation to the chosen benefactor in each Efficiency condition. Specifically, we defined the “Relative Self‐payoff” as ‘$\frac{the\;amount\;reserved\;for\;oneself}{\left(the\;amount\;reserved\;for\;oneself+efficiency\;*\;the\;allocation\;to\;the\;benefactor\right)}$’, representing the relative concern for self‐interest in relative to the concern for the chosen benefactor's interest. For one thing, we compared the Relative Self‐payoff when participants chose the Strategic Benefactor in the A1S3 and A1S1 conditions using the Mann‐Whitney U Test (see *Methods*), which demonstrated how the increased efficiency of the Strategic Benefactor modulated the Relative Self‐payoff. Results showed that the Relative Self‐payoff decreased (but not increased) as the efficiency of reciprocating the Strategic Benefactor increased (*Z* = 1.957, *p* = 0.050; Figure 2C; and Tables S2 and S3, Supporting Information). For another, we compared the Relative Self‐payoff when participants chose the Altruistic Benefactor in A3S1 and A1S1 conditions, which demonstrated how increased efficiency of Altruistic Benefactor modulated the Relative Self‐payoff. No significant difference in the Relative Self‐payoff was observed between the A3S1 and A1S1 conditions (*Z* = ‐1.550, *p* = 0.121; Figure 2D; and Tables S2 and S3, Supporting Information). These results were replicated in the additional behavioral with independent sample (see *Supplementary Results* and Figure S3, Supporting Information).

These results, along with those reported above, further suggested that the reciprocal efficiency influenced participants' reciprocal partner choices by modulating their concern for the benefactor (the trade‐off between feelings of communal concern and obligation), rather than the concern for self‐interest.

### 2.4 Verifying the Modulation Effect of Efficiency on the Trade‐Off Between Communal and Obligation Feelings Using Cross‐Study Neural Expressions

**Figure 3:**
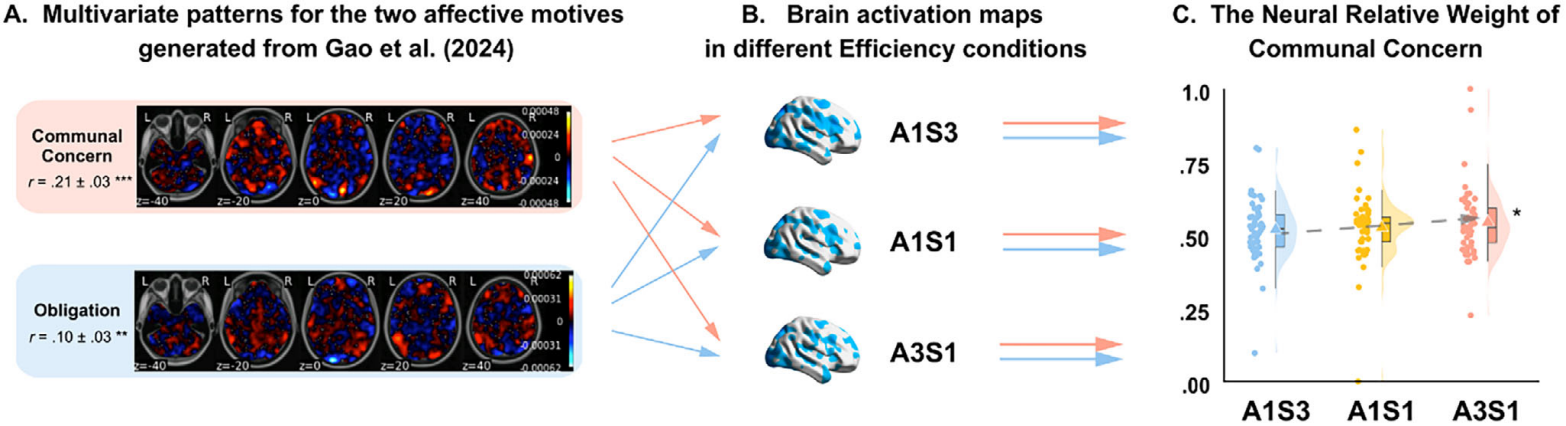
Cross‐study neural expressions of communal concern and obligation. A) Multivariate patterns generated from the previous study. Whole‐brain multivariate pattern maps for the processing of communal concern (Pink), and the processing of obligation (Blue), which were established by our recent research.^[^ ^47^ ^]^ B) Brain activation maps in three Efficiency conditions. For each participant, we computed the dot‐product of the contrast map for each Efficiency condition (A1S3, A1S1, and A3S1) with the whole‐brain multivariate pattern map for the processing of communal concern and obligation, separately. C) The neural relative weight of Communal Concern in three Efficiency conditions. Similar to the behavioral analysis, we also defined “the neural relative weight of Communal Concern” as $\frac{\left|\mathrm{the}\;\mathrm{neural}\;\mathrm{predicted}\;\mathrm{processing}\;\mathrm{of}\;\mathrm{communal}\;\mathrm{concern}\right|}{\left(\left|\mathrm{the}\;\mathrm{neural}\;\mathrm{predicted}\;\mathrm{processing}\;\mathrm{of}\;\mathrm{communal}\;\mathrm{concern}|+|\mathrm{the}\;\mathrm{neural}\;\mathrm{predicted}\;\mathrm{processing}\;\mathrm{of}\;\mathrm{obligation}\right|\right)}$, which reflected the neural trade‐off between communal and obligation feelings when participants made reciprocal partner choices. Direct comparison revealed a significant linear increase of the neural relative weight of Communal Concern as the increase of the reciprocal efficiency of the Altruistic Benefactor (i.e., from A1S3 to A1S1 and A3S1). Each dot represents one participant (*n* = 50). Significance: * *p* < 0.05, ** *p* < 0.01, and *** *p* < 0.001.

At the behavioral level, we observed the modulation effect of efficiency on the trade‐off between communal and obligation feelings behind reciprocal partner choices. We further verify this finding by conducting cross‐study neural expressions for the processing of communal and obligation feelings using the whole‐brain multivariate pattern maps for these two emotional motives established by our recent research^[^ ^47^ ^]^ (see *Methods*). Similar to the behavioral data analysis, we calculated the neural relative weight of Communal Concern, but here we used neural expression values instead of *β* values. We defined “the neural relative weight of Communal Concern” as $\frac{\left|\mathrm{Neural}\;\mathrm{Value}\;\mathrm{communal}\;\mathrm{factor}\right|}{\left(|\mathrm{Neural}\;\mathrm{Value}\;\mathrm{communal}\;\mathrm{factor}|+|\mathrm{Neural}\;\mathrm{Value}\;\mathrm{obligation}\;\mathrm{factor}|\right)}$ which reflected the neural trade‐off between communal and obligation feelings when participants made reciprocal partner choices. Consistent with our behavioral results (Figure 2B), a one‐way (Efficiency: A1S3, A1S1, A3S1) repeated‐measures analysis of variance (ANOVA) on the neural relative weight of Communal Concern revealed a significant main effect of Efficiency (Greenhouse‐Geisser correction: *F*_(1.63, 79.98)_ = 3.355, *p* = 0.049, *η_p_^2^* = 0.064, permutation *p* < 0.001; **Figure** **3**C). The direct comparison revealed a significant linear increase of the neural relative weight of Communal Concern as the increase of the reciprocal efficiency of the Altruistic Benefactor (i.e., from A1S3 to A1S1 and A3S1; *F*_(1,49)_ = 6.707, *p* = 0.013, *β* = 0.012, permutation *p* = 0.007; Figure 3C). To be more specific, the neural relative weight of Communal Concern was significantly higher in the A3S1 condition, compared with the A1S3 condition (*mean difference* = 0.025, *Standard Error* = 0.009, *95%CI* = [0.005, 0.044], *p* = 0.013); although the difference between A3S1 condition and A1S1 condition (*mean difference* = 0.018, *Standard Error* = 0.012, *95%CI* = [‐0.006, 0.042], *p* = 0.133), and that between A1S1 condition and A1S3 condition (*mean difference* = ‐0.007, *Standard Error* = 0.008, *95%CI* = [‐0.022, 0.009], *p* = 0.403), did not reach significance. Analysis on the original neural expression values for Communal Concern and Obligation yielded a similar pattern of results (see *Supplementary Results* and Figure S6, Supporting Information). These results verified the modulation effect of efficiency on the trade‐off between communal and obligation feelings at the neural level.

### 2.5 Identifying the Neural Bases Underlying the Influence of Reciprocal Efficiency

**Figure 4:**
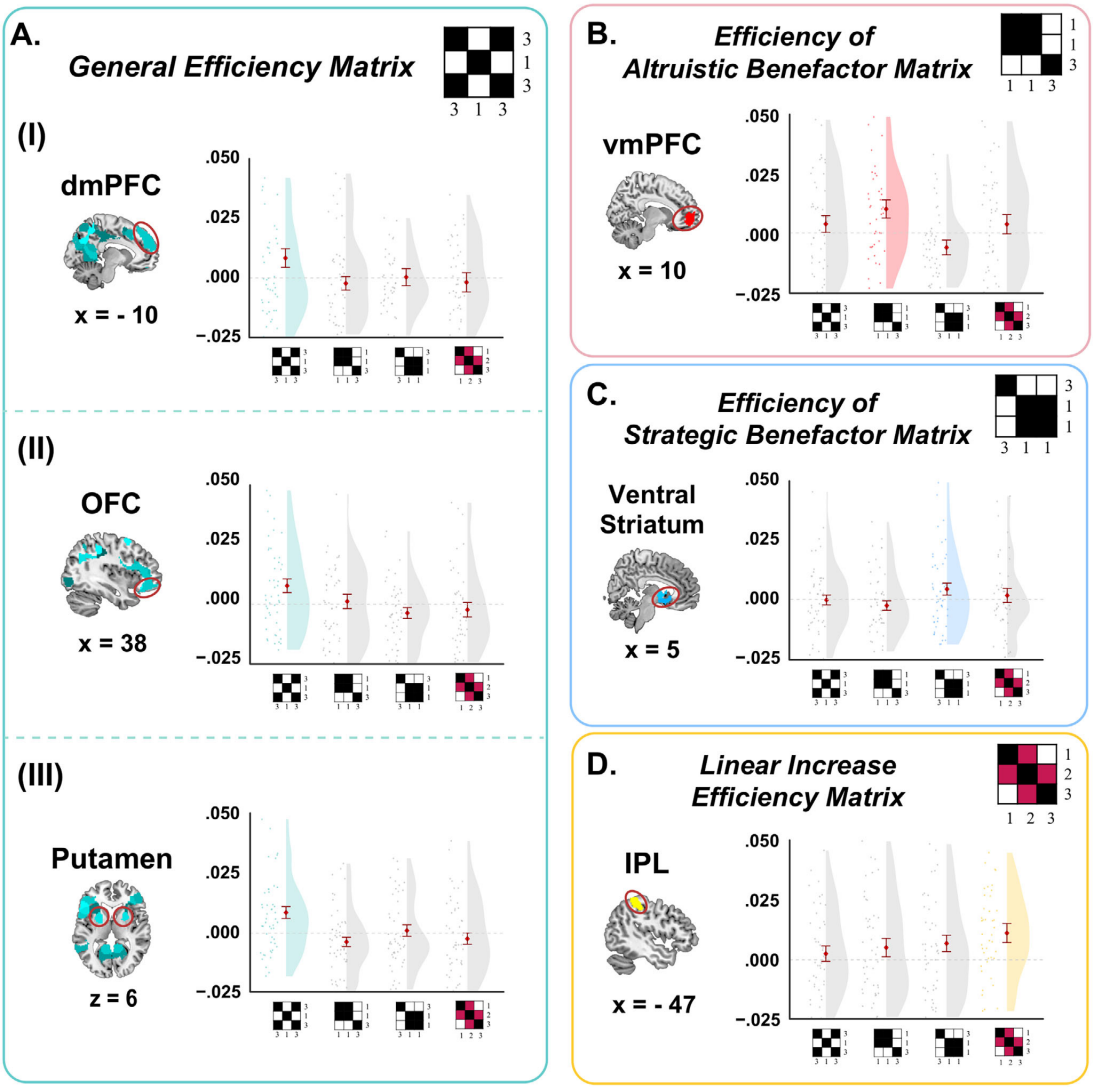
Intra‐subject RSA results. A) Brain regions that are involved in the general processing of efficiency, independent of the type of the benefactor. Results revealed the involvements of the dorsomedial prefrontal cortex (dmPFC) (I), the lateral orbitofrontal cortex (LOFC) (II), and putamen (III). B,C) Neural activities that responded specifically to the efficiency of the Altruistic Benefactor and the Strategic Benefactor, respectively. The increased reciprocal efficiency of the Altruistic Benefactor was associated with enhanced activation in the ventromedial prefrontal cortex (vmPFC); while the increased reciprocal efficiency of the Strategic Benefactor was associated with enhanced activation in the ventral striatum (VS). D) Brain regions that are related to the processing of the linear effect of efficiency on reciprocal partner choices and the underlying emotional motives. Results revealed significant activation in the inferior parietal lobe (IPL). Data are presented as mean values ± SEM. Each dot represents the average beta value (*β*) for each participant (*n* = 50 participants). Significance: * *p* < 0.05; *** *p* < 0.001.

Although multivariate pattern maps based cross‐study neural expressions enable us to examine the involvements of the processing of two emotional motivations in different reciprocal efficiency conditions at the whole‐brain level, this analysis cannot identify the specific brain regions implicated in this process. Therefore, we then applied intra‐subject multivariate representational similarity analyses (RSAs) to identify the specific brain regions underpinning the effects of efficiency on reciprocal partner choices, using an a priori 200‐parcel whole‐brain parcellation.^[^ ^26^, ^110^ ^]^ Specifically, within each parcel, we created a dissimilarity matrix for neural activations (the Parcel Dissimilarity Matrix) using pairwise correlation dissimilarity between each pair of the 27 conditions (3 Efficiency conditions × 9 levels of Benefactor's cost). We then estimated the association between the Parcel Dissimilarity Matrix and each of the four cognitive dissimilarity matrices that characterized different cognitive processes: the general processing of reciprocal efficiency, the processing of Altruistic Benefactor's efficiency, the processing of Strategic Benefactor's efficiency, and the processing related to the effect of efficiency on reciprocal partner choices (see *Methods*). Results demonstrated distinct neural bases underlying these four types of cognitive processes (**Figure** **4**).

Specifically, first, we applied the General reciprocal efficiency Matrix (A1S3, A1S1, and A3S1 were coded as 3, 1, and 3) to identify the brain regions that are involved in the general processing of reciprocal efficiency, independent of the type of the benefactor (Figure 4A). Results revealed the involvements of the dorsomedial prefrontal cortex (dmPFC; *coefficient* = 0.008, *Standard Error* = 0.002, *Z* = 3.366, *95%CI* = [0.003, 0.013], FDR corrected *p* = 0.006), which is associated with theory of mind,^[^ ^9^, ^47^, ^111^, ^112^ ^]^ and the lateral orbitofrontal cortex (LOFC; *coefficient* = 0.008, *Standard Error* = 0.002, *Z* = 3.591, *95%CI* = [0.004, 0.012], FDR corrected *p* < 0.001), which is related to reward value representation.^[^ ^9^, ^60^, ^113^, ^114^, ^115^, ^116^, ^117^ ^]^ Importantly, we also found significant activation in putamen (*coefficient* = 0.009, *Standard Error* = 0.002, *Z* = 4.714, *95%CI* = [0.005, 0.012], FDR corrected *p* < 0.001), which was found to directly respond to efficiency processing in a resource allocation task.^[^ ^98^ ^]^

Second, the Efficiency of Altruistic Benefactor Matrix (A1S3, A1S1, and A3S1 were coded as 1, 1, and 3) and the Efficiency of Strategic Benefactor Matrix (A1S3, A1S1, and A3S1 were coded as 3, 1, and 1) were applied to reveal the neural activities that responded specifically to the efficiency of the Altruistic Benefactor and the Strategic Benefactor, respectively (Figure 4B). Results demonstrated that the increased reciprocal efficiency of the Altruistic Benefactor was associated with enhanced activation in the ventromedial prefrontal cortex (vmPFC; *coefficient* = 0.010, *Standard Error* = 0.003, *Z* = 3.598, *95%CI* = [0.005, 0.015], FDR corrected *p* < 0.001), an area in the default mode network that has been linked to gratitude,^[^ ^47^, ^51^, ^52^, ^118^ ^]^ positive social value,^[^ ^119^, ^120^ ^]^ and kind intention;^[^ ^9^, ^52^, ^121^ ^]^ while the increased reciprocal efficiency of the Strategic Benefactor was associated with enhanced activation in the ventral striatum (VS; *coefficient* = 0.004, *Standard Error* = 0.004, *Z* = 2.320, *95%CI* = [0.001, 0.008], FDR corrected *p* = 0.040), which plays a dominant role in reward processing.^[^ ^9^, ^21^, ^51^, ^60^, ^106^, ^122^, ^123^, ^124^ ^]^

Third, to search for the brain regions of the processing related to the linear effect of efficiency on reciprocal partner choices (Figure 2A) and the underlying emotional motives (Figure 2B), we built the Linear Increase Effect Matrix (A1S3, A1S1, and A3S1 were coded as 1, 2, and 3), which corresponded to the pattern of linear increase across the A1S3, A1S1, and A3S1 conditions that observed in above results for reciprocal partner choices and the relative weight of Communal Concern (Figure 4B). We observed significant associations in the inferior parietal lobe (IPL; *coefficient* = 0.011, *Standard Error* = 0.003, *Z* = 3.653, *95%CI* = [0.003, 0.017], FDR corrected *p* < 0.001), which was linked to calculation abilities, social cognition, and perspective‐taking in previous studies.^[^ ^47^, ^68^, ^125^, ^126^, ^127^, ^128^, ^129^, ^130^, ^131^, ^132^, ^133^, ^134^ ^]^

## 3 Discussion

In real‐world societies, the application of direct reciprocity and partner choice principles is integral to addressing diverse challenges, ranging from world‐wide responses to extreme climatic disruptions, corporate fund‐raising, to mutual assistance among citizens. Consequently, elucidating the cognitive and neural mechanisms underlying individual decision‐making in these contexts holds significant theoretical and practical importance. However, within the field of cognitive neuroscience, research on reciprocity has predominantly adopted a partner control perspective,^[^ ^7^, ^8^, ^9^, ^10^ ^]^ largely overlooking the critical role of partner choice. Furthermore, existing studies on partner choice primarily employ evolutionary simulations^[^ ^135^, ^136^, ^137^, ^138^, ^139^ ^]^ or social psychological investigations,^[^ ^34^, ^140^, ^141^ ^]^ with a notable scarcity of cognitive neuroscience research. Our study contributes to addressing both of these two gaps, providing novel evidence for understanding cooperation formation at the level of individual decision mechanisms, and establishing a theoretical foundation for related real‐world decision‐making and practical applications.

By combining an interpersonal game with fMRI multivariate neural expressions and RSA, we unveil the cognitive and neural mechanisms through which reciprocal efficiency recalibrates communal concern and obligation in the social dilemma of partner choice in direct reciprocity. Behavioral results demonstrate that reciprocal efficiency robustly shifts preferences between altruistic and strategic benefactors. Evidence from both behavioral analyses and fMRI multivariate neural expressions suggest that, these reciprocal decisions are not merely rational optimization, but reflect deeper cognitive calculus balancing the feelings of communal concern and obligation. When reciprocating altruistic benefactors yielded higher efficiency, participants prioritized communal concern (gratitude/guilt) over obligation, thus favoring altruistic benefactors, while when reciprocating strategic benefactors yielded higher efficiency, participants prioritized obligation over communal concern, thus favoring strategic benefactors. RSA on fMRI data further revealed both differential and similar neural representations underlying these two directions of the facilitative effects of reciprocal efficiency: 1) the former was associated with the neural representations in the vmPFC, while the latter was associated with the neural representations in the VS; 2) the dmPFC, LOFC, and putamen were linked to the general processing for both, independent of the type of the benefactor; and 3) the IPL encoded the linear trade‐off between communal and obligation concerns, underscoring the computational nature of efficiency‐driven recalibration.

Previous investigations into the psychological and neural underpinnings of partner choice have predominantly centered on how individuals formulate decisions when presented with diverse partner‐related information,^[^ ^38^, ^142^, ^143^, ^144^, ^145^, ^146^, ^147^ ^]^ or how they acquire partner traits to subsequently execute partner choices.^[^ ^45^, ^142^, ^148^, ^149^, ^150^, ^151^ ^]^ Within these research frameworks, participants generally adhere to relatively well‐defined criteria for assessing the qualities of partners, such as their propensity for generosity,^[^ ^146^, ^152^, ^153^ ^]^ commitment to fairness,^[^ ^30^, ^154^ ^]^ degree of warmth,^[^ ^45^, ^155^ ^]^ or level of competence.^[^ ^31^, ^32^, ^41^ ^]^ In contrast to these existing studies, the present research spotlights a prevalent yet markedly underexplored social‐emotional dilemma in real‐life contexts: when confronted with an altruistic benefactor without requirement for reciprocation and a strategic benefactor who expects reciprocation, it is deemed socially appropriate for the beneficiary to reciprocate to either type of benefactor; thus, this decision‐making of partner choice lacks a definitive standard answer. Under such circumstances, our findings reveal that reciprocal efficiency can functions as a pivotal “weight” on the dilemma “scale”, dynamically recalibrating the social‐emotional motivation weights that underlie reciprocal partner choice. This mechanism may hold significant adaptive value. As beneficiaries, individuals may be compelled by their limited resources to reassess or even forgo certain emotional concerns associated with reciprocity in order to optimize reciprocal efficiency, as indiscriminate generosity may deplete critical reserves.^[^ ^156^ ^]^

It is important to highlight that, on the one hand, prior economic research has extensively documented the crucial role of efficiency in fostering cooperative behaviors.^[^ ^84^, ^85^, ^87^, ^90^, ^91^, ^95^ ^]^ However, these studies have predominantly overlooked the social‐emotional motivations that drive decision‐making, particularly in the context of reciprocal decisions. On the other hand, psychological and cognitive neuroscience studies have elucidated the dual emotional motivations that support reciprocity,^[^ ^6^, ^47^, ^48^, ^49^, ^50^, ^157^ ^]^ but have largely neglected the influence of efficiency on these processes. From this view, the current study makes a significant contribution by bridging a longstanding divide between economic models of efficiency‐driven cooperation and psychological theories of social emotion‐guided reciprocity, offering novel insights into the understanding of human cooperation. On the other hand, prior work posits efficiency as a standalone driver for cooperation that independent form other motivations.^[^ ^97^, ^98^, ^99^, ^100^, ^101^, ^102^, ^103^ ^]^ However, both our findings from behavioral regression analysis and cross‐study neural expressions analysis reveal that, efficiency can serve as a meta‐cognitive moderator of social/moral emotional motivations behind reciprocal choices. Simultaneously, the analysis on the amounts of reciprocity to the chosen benefactors indirectly indicates that participants’ relative concerns for self‐interest did not increase with reciprocal efficiency. This indicates, moral considerations and efficiency considerations are not orthogonal but interactively shape decisions, since participants did not merely make trade‐off between “the right” or “the moral”,^[^ ^98^, ^158^ ^]^ but dynamically adjusted their moral calculus based on how effectively their actions maximized relational or material gains. Our findings provide important insights for future research on efficiency in social decision‐making, suggesting its potential interaction with moral motivations should be included in decision‐making models, in addition to being weighed as a separate motivation against other motivations.

At the neural level, our recent study has employed a partner‐control perspective and revealed the neural bases of how communal concern and obligation generate and influence the beneficiary's amount of reciprocity to one specific benefactor.^[^ ^47^ ^]^ Here, our cross‐study neural expressions based on multivariate pattern maps established by this recent work provide whole‐brain level evidence for the recalibration of reciprocal efficiency on communal concern and obligation behind reciprocal choices. By applying RSA, we then identify the neural representations underpinning these processes, which are consistent with the findings of our recent study on partner‐control‐based reciprocity. For instance, this recent study highlighted the vmPFC as crucial for processing communal concern, a region linked to gratitude,^[^ ^47^, ^51^, ^52^, ^118^ ^]^ positive social value,^[^ ^119^, ^120^ ^]^ and kind intention.^[^ ^9^, ^52^, ^121^ ^]^ We found that this same region showed enhanced representation when participants faced the altruistic benefactor, a context that elicits stronger communal concern. Similarly, while this recent study revealed the role the dmPFC in obligation, a region linked to theory of mind and intention inference,^[^ ^9^, ^47^, ^112^ ^]^ we found its activity was also more strongly represented when participants faced the strategic benefactor, where obligation considerations are heightened. Finally, while the IPL was found to encode the linear trade‐off between communal and obligation motives and drives adjustments in reciprocal behavior in the current study, this region was identified in the recent study as integral for integrating motives to reach a reciprocal decision^[^ ^47^ ^]^ and in other studies as a hub for cost‐benefit integration and calculation.^[^ ^68^, ^126^, ^128^, ^129^, ^133^, ^134^ ^]^ The cross‐study consistency at both whole‐brain and ROI levels attests to the robustness of our results.

More importantly, we provide advanced evidence showing that the enhanced consideration for communal concern when the altruistic benefactor's efficiency increased, and the enhanced consideration for obligation when the strategic benefactor's efficiency increased, were supported by both shared and benefactor‐intention‐specific neural bases. On the one hand, by comparing high‐ and low‐ reciprocal efficiency conditions, regardless of the type of the benefactors, we found the activation of putamen for general reciprocal efficiency processing, which is line with consistent with previous findings for the efficiency processing resource allocation.^[^ ^98^ ^]^ Moreover, we identify the involvements of LOFC and dmPFC. The involvement of LOFC, which has been revealed to play crucial roles in value comparison and social norm internalization,^[^ ^9^, ^60^, ^113^, ^114^, ^115^, ^116^, ^117^ ^]^ suggests the beneficiaries may need to internalize considerations of reciprocal efficiency into their intrinsic social emotional motivations toward the benefactors, in order to adjust reciprocal behaviors. In contrast to our recent findings on partner‐control‐based reciprocity, which highlighted the role of the dmPFC in obligation processing but not in communal processing, the current study reveals its involvement in efficiency‐driven increases of both communal and obligation considerations during reciprocal partner choice. Given the important role of dmPFC in theory of mind and intention inference,^[^ ^9^, ^47^, ^112^ ^]^ we argue that this finding is consistent with previous evidence suggesting that the impact of intention inference on partner choice is even more pronounced than in partner control studies.^[^ ^30^, ^34^, ^46^ ^]^ We speculate that evaluating varying reciprocal efficiency may require beneficiaries to place more cognitive demands on inferring the resulting reactions of the benefactors and the influences of reciprocal partner choice on social the relationships. Thus, the dmPFC may support not only the inference of strategic intentions in obligation but also, more broadly, the prediction of others' reactions and the future of social relationships in high‐efficiency scenarios.^[^ ^9^, ^47^, ^112^ ^]^

On the other hand, we found that higher reciprocal efficiency was associated with enhanced activation in the vmPFC for altruistic benefactors and the ventral striatum (VS) for strategic benefactors. We posit two possible interpretations. First, this may reflect a dissociation of intrinsic motives: the vmPFC, a core region for communal concern,^[^ ^118^, ^119^, ^120^, ^121^ ^]^ may reflect the adjustment of intrinsic social‐emotional motivations, whereas the VS, a region for reward anticipation but not specifically linked to communal or obligation motives, may reflect a more reward‐driven process.^[^ ^9^, ^21^, ^106^, ^122^, ^123^, ^124^ ^]^ When facing strategic benefactors, beneficiaries may focus more on calculating explicit expected rewards to maximize utility in this strategic interaction. A second possibility is that both the vmPFC and VS are implicated in general valuation processes.^[^ ^9^, ^159^, ^160^, ^161^, ^162^ ^]^ From this perspective, our results might indicate that general efficiency valuation of efficiency may implement through different valuation systems depending on the benefactor's intention. Future research with refined designs is needed to dissociate the processing of efficiency from that of motivational weights to test these two mechanistic explanations.

These partner intention‐specific neural bases offer important insights for future research and practical applications. On the one hand, these findings provide a cognitive‐level theoretical mechanism for future studies on the formation of cooperation applying evolutionary game theory,^[^ ^138^, ^139^, ^163^, ^164^ ^]^ suggesting that the design of evolutionary models needs to incorporate specificity for the partner's intentions and the decision‐makers consideration of social‐emotional motivations. On the other hand, our findings indicate that governments and corporations can devise distinct reciprocal efficiency incentive policies to enhance and solidify specific types of social relationships in different cooperative contexts. For example, in scenarios where transactional relationships among members are desired,^[^ ^165^, ^166^, ^167^ ^]^ such as in corporations, managers can enhance the efficiency with which individuals reciprocate benefactors with transactional expectations, in order to strengthen transactional relationships and expand material benefits within the group. In contrast, in contexts where members need to establish communal relationships that provide mutual care and favor sincerely and voluntarily,^[^ ^56^, ^63^, ^143^ ^]^ such as families or sports teams, it is necessary to enhance the efficiency with which individuals reciprocate altruistic benefactors. Our study also indicates that investors need to distinguish the reciprocity preferences employed by entrepreneurs and appropriately enhance their reciprocal efficiency to obtain effective returns from their investments promptly. These insights provide important implications for future policy‐making related to investment, management, and governance.

The current study has several potential limitations, which are important to acknowledge. First, gratitude and guilt frequently co‐occur in help‐receiving contexts and exert analogous effects in promoting reciprocity.^[^ ^157^, ^168^, ^169^ ^]^ Previous research has thus conceptualized both emotions as reflecting the beneficiary's concern for the benefactor arising from responsiveness to the benefactor's kindness, and collectively termed them “communal concern” behind reciprocal behavior.^[^ ^47^, ^55^, ^56^ ^]^ The results of current study replicated previous observations, with factor analysis grouping gratitude and guilt into a single communal motivation factor. Building on this, we revealed how reciprocal efficiency modulates the trade‐off between communal and obligation motives in partner choice. That said, while research on guilt in help‐receiving contexts remains limited, studies on interpersonal transgressions suggest that guilt, as a negative emotion, can promote prosocial behavior but may also trigger adverse effects such as social avoidance.^[^ ^60^ ^]^ Whether guilt in response to receiving help carries similar negative consequences, and how it might influence reciprocal partner choice, remains an open question. However, the strong co‐occurrence of gratitude and guilt in such contexts makes it difficult to dissociate their unique effects. For this reason, our interpersonal task was designed to capture the overall influence of communal motivation rather than to explicitly differentiate guilt from gratitude, which limits our ability to address these nuanced questions. Future studies are needed to develop experimental designs or analytical approaches capable of disentangling the distinct contributions of gratitude and guilt in reciprocal decision‐making.

Second, as an initial investigation into reciprocal partner choice, the current study employed a well‐controlled experimental design. Its primary aim was to dissociate and examine the causal roles of two core variables (i.e., reciprocal efficiency and social‐emotional motives) in shaping partner choice, while minimizing the influence of confounding factors. By simplifying the social scenario in the laboratory (e.g., by making benefactor's intentions and reciprocal efficiencies explicit), we were able to demonstrate, for the first time at both the behavioral and neural levels, that reciprocal efficiency considerations systematically recalibrate the weighting of social‐emotional motivations to guide reciprocal partner choice. This constitutes a necessary foundational step toward understanding more complex real‐world settings associated with noisy naturalistic data. We believe the core cognitive principle revealed here is universally relevant. However, the relative weights of these factors and the resulting behavioral outcomes may vary depending on the real‐world complex factors, such as how explicitly or implicitly benefactor intentions and efficiencies are perceived.^[^ ^13^, ^91^, ^170^ ^]^ Building on the present findings, future research employing methods with higher ecological validity (e.g., field experiments or virtual reality) can help verify and refine the cognitive framework established in this study.

In conclusion, the current study bridges the gap between the two crucial mechanisms of cooperation, namely direct reciprocity and partner choice. Focusing on two key determinants of reciprocity—social‐affective motivations and reciprocal efficiency—our findings illuminate the cognitive processes underlying how individuals prioritize reciprocal partner when receiving help from multiple benefactors. We demonstrate that the efficiency‐driven reciprocal decisions are not purely rational but balance intention‐specific neurocognitive trade‐offs between social‐emotional motives of communal concern (gratitude/guilt represented in ventromedial prefrontal cortex) and the sense of obligation (represented in ventral striatum), through the computational integration in the inferior parietal lobe. These findings challenge traditional models isolating efficiency from morality, demonstrating their synergy in cooperation, offering novel insights into future research on cooperation and practical applications.

## 4 Experimental Section

### Participants

Fifty participants (25 females; mean age = 20.92 ± 2.02 years) were recruited from Peking University, China, for the fMRI experiment. No participant was excluded from the data analysis due to excessive head movements (> 3 mm of locomotion or 3 degrees of rotation). An additional 37 participants from East China Normal University, China, were recruited for an additional behavioral experiment (19 females; mean age = 22.08 ± 2.03 years). All participants were right‐handed with normal or corrected‐to‐normal vision, and none reported any history of neurological or psychological disorders. All experiments were conducted following the Declaration of Helsinki and were approved by the Ethics Committee of the School of Psychological and Cognitive Sciences, Peking University (2019‐03‐04), and the University Committee on Human Research Protection (UCHRP), East China Normal University (HR2‐0233‐2022). Informed written consent was obtained from each participant before the experiment.

### Experimental Procedure—Overview

The fMRI experiment consisted of 3 sessions. In Session 1 (pain calibration), each participant was first informed that they would later play an interpersonal task concerning pain stimulation and thus a calibration of pain level was performed before the task. In Session 2, the participant performed an interpersonal task (the main task) as the beneficiary in the fMRI scanner, in which they received help from different types of benefactors and decided to whom and how much to reciprocate. In Session 3 (subjective ratings), participants were instructed to recall and rate their feelings and beliefs toward the benefactors in the behavioral lab. All stimuli were presented using PsychToolBox 3.0.12 (www.psychtoolbox.org) in Matlab 2018a (Mathworks, Natick, MA, USA).

### Experimental Procedure—Pain Calibration

The procedure of this session was set regarding previous studies on social emotions.^[^ ^47^, ^51^, ^52^, ^59^, ^171^, ^172^ ^]^ An intra‐epidermal needle electrode was positioned on the left wrist of all participants to deliver cutaneous electrical stimuli. An initial stimulus train comprised 8 pulses (0.2 mA intensity, 0.5 ms duration each), delivered with a 10‐ms inter‐pulse interval. Subsequently, stimulus intensity was iteratively adjusted upwards for each participant until they reported moderate pain, corresponding to a rating of 6 on an 8‐point scale (where 1 denoted no pain and 8 represented intolerable pain). Participants consistently perceived the pulse sequence as a single, integrated percept rather than discrete shocks. This calibration procedure ensured the final stimulus intensity consistently evoked a subjectively moderate level of discomfort.

### Experimental Procedure—The Interpersonal Task

The current interpersonal task was adapted from the recent study,^[^ ^47^ ^]^ which adopted a perspective of partner control, and aimed to reveal how beneficiaries generate, weigh and integrate the two emotional motivations of communal concern (gratitude and guilt) and obligation, in order to make decisions of how much to reciprocate to a specific benefactor with altruistic or strategic intention. In each round of the interpersonal task in this recent study, participants were instructed that they would receive 20 s of pain stimulation and were randomly paired with a different anonymous same‐gender co‐player (benefactor), who had been endowed with 20 yuan (around USD 2.8) and made a decision about how much to spend from this endowment to reduce the duration of pain experienced by the participant during a separate lab visit. After seeing the benefactor's decision, the participants decided how much of their own 25 yuan endowment (≈$3.5 USD) they wanted to allocate to the benefactor as reciprocity for their help. Participants' beliefs about the benefactors' intentions were manipulated by providing additional information regarding the benefactors' expectations of reciprocation. Each participant was instructed that before making decisions, some benefactors knew that the participant would be endowed with 25 yuan and could decide whether to allocate some endowments to them as reciprocity (i.e., Repayment possible condition), whereas the other benefactors were informed that the participant had no chance to reciprocate after receiving help (i.e., Repayment impossible condition). In fact, participants could reciprocate in both conditions during the task. After the task, all trials were displayed again in a random order and participants recalled the perceived benefactors’ intentions, as well as their feelings of indebtedness, obligation, guilt, and gratitude in response to the help they received for each trial. Using this task, this recent study provided consistent evidence at behavioral, cognitive computational and neural levels showing that, the beneficiary's two emotional motivations of communal concern (gratitude and guilt) and obligation arise from the apparels of the benefactors’ altruistic and strategic intentions, respectively, and then were weighed and integrated to generate the decisions of how much to reciprocate to a specific benefactor.

To address the absence of the task for partner choice in direct reciprocity and to answer the proposed scientific questions, the current study introduced a critical modification on the task: participants now faced two benefactors with different intentions simultaneously instead of one, and made decisions of choosing one of them to reciprocate. Although the present study and the recent research^[^ ^47^ ^]^ differ fundamentally in the research question and the type of reciprocal choice participants make, they share a similar experimental foundation, i.e., both involving the generation and weighting of beneficiary's emotions of gratitude, guilt and obligation after receiving help. Therefore, the recent research^[^ ^47^ ^]^ provides a substantial foundation for validating the efficacy and appropriateness of emotion induction, measurement, and analysis methodologies employed in the present study.

Specifically, in the current study, after the pain titration, participants were instructed to read the instructions for the interpersonal task. They were informed that the task consisted of two roles: Player A (Benefactor) and Player B (Beneficiary). Some participants had come to the lab as benefactors and made decisions about whether and how to help Player Bs. The participants would act as the role of beneficiaries, making reciprocal decisions based on the benefactors’ previous decisions.

Then, each participant was informed that they had to receive two 20 s pain stimulations with an intensity of 6 in each trial, and each benefactor: 1) had come to the lab before the participant, 2) had been given an endowment of 20 yuan (around USD 2.8), and 3) had decided whether, and how much, to spend from their endowment to reduce the duration of the pain stimulation the participants were assigned to, with each benefactor corresponding to one of the two stimulations. (i.e., benefactor's cost). With a conversion rate of 0.8, the benefactor's expenditure was directly proportional to the reduction in the participant's pain duration. In other words, the more the benefactor spent, the shorter the duration of the participant's pain experience. The maximum pain reduction was capped at 16 s (i.e., when the benefactor spent 20 yuan) to ensure that participants would not experience the extreme scenarios of “no pain”. Unbeknownst to the participants, all decisions of the benefactors were predetermined by the computer program.

In each trial (Figure 1B), the participant was matched with two anonymous benefactors of the same gender and was informed that the two benefactors in each round were different from those in any other trials and would interact with the participant only once during the task. Before making decisions, one of the two benefactors was informed that the participant would be endowed with 25 yuan (around USD 3.5) and could decide whether to allocate some endowments to them as reciprocity, while the other benefactor was informed that the participant had no chance to reciprocate after receiving help. Based on the findings of the recent study,^[^ ^47^ ^]^ the participant would perceive the former benefactor's help as a strategic action with the expectation for repayment; therefore, this type of benefactor was referred to as the “Strategic Benefactor” in the following. Meanwhile, the participant would perceive the later benefactor's help as an altruistic action without expectation for repayment; therefore, this type of benefactor was referred to as the “Altruistic Benefactor” in the following. The participant was instructed that these two benefactors had decided to spend the same amount of money to help the participant (i.e., the same benefactor's cost) and that now the participant was endowed with 25 yuan but could only choose one of them to reciprocate. Moreover, the efficiency of reciprocating to the two benefactors might vary, with this reciprocal efficiency being 1 or 3, as determined randomly by the computer program. With reciprocal efficiency being 1, for every 1 yuan the participant reciprocated, the benefactor would receive 1 yuan; with the reciprocal efficiency being 3, for every 1 yuan the participant reciprocated, the benefactor would receive 3 yuan. In simpler terms, the extra reward that the benefactor received would be the participant's amount of reciprocity multiplied by the corresponding reciprocal efficiency. To note, participants were instructed that all the benefactors made their decisions without knowledge of the reciprocal efficiency. Having learned about the above rules, the participant should decide which benefactor they would like to reciprocate and then how much they allocate to this benefactor as reciprocity in each trial.

Each trial began by informing the participant that the program had randomly chosen two benefactors for the current trial (Grouping Period, 1–3 s). Then, information regarding the two benefactors’ spending and the corresponding amount of pain reduction for the participant was presented (Outcome Period, 3–5 s). Subsequently, participants would see the two benefactors’ blurred photos and IDs, presented on the left and right sides of the screen, respectively. The information that the benefactor knew the participant could or could not repay, as well as the reciprocal efficiency of each benefactor, were given underneath the corresponding photo. The participant had to choose one benefactor to reciprocate (i.e., reciprocal partner choice) by pressing the left or the right button (Choice Period, < 8 s). A box would appear around the selected benefactor and last for 1 s (Choice Feedback, 1 s). At the end of each trial, the participant was endowed with 25 yuan and decided how much to allocate to the chosen benefactor as reciprocity (Allocation Period, < 8 s, continuous choice from 0 to 25, step of 1 yuan). For fMRI signal deconvolution, before and after the Choice Period, a fixation cross was presented for a variable interval ranging from 1 to 5 s.

At the end of the experiment, one trial in the interpersonal task was randomly selected to be realized. The participant received pain stimulation in this trial. The participant's final payoff was the amount of endowment the participant left for him/herself in the chosen trial. The final payoff of the chosen benefactor was the amount of endowment this benefactor left plus the product of the reciprocal efficiency and the amount of endowment the participant allocated to him/her. Participants were informed of this arrangement before the execution of the experiment.

There were three reciprocal efficiency conditions: 1) higher reciprocal efficiency of the Strategic Benefactor (A1S3): the efficiency of the Altruistic Benefactor was 1, while that of the Strategic Benefactor was 3; 2) equal reciprocal efficiency of the two benefactors (A1S1): both benefactors had an efficiency of 1; 3) higher reciprocal efficiency of the Altruistic Benefactor (A3S1): the efficiency of the Altruistic Benefactor was 3, while that of the Strategic Benefactor was 1. Moreover, to increase the variability of participants’ social‐emotional responses in the task, the Benefactor's cost was parametrically manipulated (including 9 levels: 4, 6, 8, 10, 12, 14, 16, 18, and 20). This formed a 3 (Efficiency: A1S3, A1S1, A3S1) × 9 (Benefactor's Cost: 4, 6, 8, 10, 12, 14, 16, 18, and 20) within‐subject design. The task consisted of 3 runs (81 trials in total) and lasted ≈27 min. Specifically, each run lasted 9 min and included 27 trials, encompassing 9 levels of the Benefactor's Cost in each of the 3 conditions of Efficiency, respectively. The trial order was randomized within each run.

### Experimental Procedure—Subjective Ratings

During this session, the participant was presented with only one benefactor, along with the corresponding information of this benefactor's cost and this benefactor knew the participant could/could not repay. To increase the variability of participants’ emotional responses, the benefactor's cost varies across 6, 10, 14, 18, and 20. Then the participant was asked to recall how much they believed this benefactor cared about them (i.e., perceived care, rated on a scale from 0 to 100, with 0 representing “not at all” and 100 representing “extremely intensive”), and how much this benefactor expected for repayment (i.e., second‐order belief, rated on a scale from 0 to 25, with each unit representing 1 yuan; rescaled to a range of 0 to 100 during data analysis). Moreover, the participant was required to recall the intensity of their feelings of gratitude, guilt, obligation, and indebtedness in response to each benefactor's help (on a scale from 0 to 100, with 0 representing “not at all” and 100 representing “extremely intense”). The order of these ratings was counter‐balanced across trials. This way of post‐experiment ratings had been proven effective in previous studies on social emotions, such as guilt and gratitude.^[^ ^47^, ^51^, ^52^, ^60^, ^113^, ^173^ ^]^ The questions for self‐reported ratings on guilt and obligation were based on previous research.^[^ ^47^ ^]^

- “How much gratitude do you feel for this benefactor's decision?” (Gratitude)
- “How much indebtedness do you feel for this benefactor's decision?” (Indebtedness)
- “How much pressure did you feel for the benefactor's expectation for repayment?” (Obligation)
- “How much guilt do you feel for this benefactor's decision?”(Guilt)
- “How much do you think this benefactor cares about you?” (Perceived care)
- “How much do you think this benefactor expected you to repay?” (Second‐order belief)

### Behavioral Analyses of the fMRI Experiment—The Effects of Efficiency and Cost on Reciprocal Partner Choices

A 3 (Efficiency: A1S3, A1S1, A3S1) × 9 (Benefactor's Cost: 4, 6, 8, 10, 12, 14, 16, 18, and 20) repeated‐measures analysis of variance (ANOVA) was conducted to test how reciprocal efficiency and the benefactor's cost modulated participants’ reciprocal partner choices, with participants’ choices encoded as the probability of choosing the Altruistic Benefactor. A Greenhouse‐Geisser correction was applied considering the violation of the sphericity assumption (similarly in the subsequent analyses). Given that mainly the effect of Efficiency was focused on and that a significant interaction between Efficiency and the Benefactor's Cost (see *Results* for details) did not found, it was opted to combine the data of all levels of the Benefactor's Cost in the subsequent analyses.

### Behavioral Analyses of the fMRI Experiment—The Influence of Efficiency on the Trade‐Off Between Communal and Obligation Feelings Underlying Reciprocal Partner Choices

As stated above, although the present study and the recent research^[^ ^47^ ^]^ differ fundamentally in the research question and the type of reciprocal choice participants make, they share a similar experimental foundation, i.e., both involving the generation and weighting of the beneficiary's emotions of gratitude, guilt and obligation after receiving help. Therefore, as manipulation checks of the inductions and measurements of emotions and appraisals, two lines of analyses were conducted on participants’ subjective ratings referring to the recent research.^[^ ^47^ ^]^ First, paired‐sample t‐tests were performed to examine whether there were differences in participants’ subjective ratings of appraisals (perceived care and second‐order belief) and emotions (gratitude, guilt, and obligation) toward Altruistic and Strategic Benefactors and whether these results replicated previous research.^[^ ^47^ ^]^ It was important to note that the values of second‐order belief was rescaled to a range of 0–100, to maintain consistency in the measurement scale range across different variables. Second, to reduce the dimensionality of data and extract the principal components of communal concern and obligation from these appraisals and emotions, Confirmatory Factor Analysis (CFA) was conducted on participants’ subjective ratings of appraisals (perceived care and second‐order belief) and emotions (gratitude, guilt, and obligation) based on the previous research,^[^ ^47^ ^]^ and obtained two main factors, namely the communal factor (*PC_communal_*) and the obligation factor (*PC_Obligation_*). The former reflected participants’ perception that the benefactor cared about their welfare and resulted in emotions of guilt and gratitude, while the latter reflected participants’ second‐order beliefs about the benefactor's expectation for repayment and the sense of obligation.

Then, to explore whether and how reciprocal efficiency modulated the contributions of feelings of communal concern (gratitude and guilt) and obligation (sense of obligation) to reciprocal partner choices, LMMs were conducted and extracted the regression coefficients (*βs*) of communal and obligation factors for predicting the probability of choosing the Altruistic Benefactor in each of the three efficiency conditions respectively. By‐participant random slopes for each fixed effect were included in each LMM. “The relative weight of Communal Concern” was defined as ‘$\frac{|\beta_{communal\;factor}|\;}{(|\beta_{communal\;factor}\left|+\right|\beta_{obligation\;factor}|)\;}$’, indicating the trade‐off between the feelings of communal concern and obligation when making reciprocal partner choices of whether choosing the Altruistic Benefactor or not. Then a one‐way (Efficiency: A1S3 vs A1S1 vs A3S1) repeated‐measures analysis of variance (ANOVA) was conducted on the relative weight of Communal Concern to explore the influence of efficiency on the trade‐off between these two affective motives.

### Behavioral Analyses of the fMRI Experiment—The Influence of Efficiency on the Relative Concern for Self‐Interest

It was also examined whether the reciprocal efficiency modulated participants’ relative concern for self‐interest. Although participants’ concerns could not estimated for self‐interest directly from their reciprocal partner choices in the current experimental design, these preferences could infer from the participants’ amounts of allocation to the chosen benefactor in each Efficiency condition. Here, the “Relative Self‐payoff” was defined as ‘$\frac{\mathrm{the}\;\mathrm{amount}\;\mathrm{reserved}\;\mathrm{for}\;\mathrm{oneself}}{\left(\mathrm{the}\;\mathrm{amount}\;\mathrm{reserved}\;\mathrm{for}\;\mathrm{oneself}\;+\;\mathrm{efficiency}\;*\;\mathrm{the}\;\mathrm{allocation}\;\mathrm{to}\;\mathrm{the}\;\mathrm{benefactor}\right)}$’, reflecting the relative concern for self‐interest in comparison to the concern for the chosen benefactor's interest. According to the experimental design, in the Allocation Period, participants could only reciprocate to the chosen benefactor. Consequently, the number of data points of the allocations varied depending on the type of benefactor participants chose, leading to unequal numbers of data points for the Relative Self‐payoff across different benefactor types. Therefore, the Relative Self‐payoff was compared when participants chose the Strategic Benefactor in the A1S3 and A1S1 conditions using the Mann‐Whitney U Test (for non‐normal data), to reveal whether and how the Relative Self‐payoff varied as the efficiency of the Strategic Benefactor increased. Similarly, the Relative Self‐payoff was compared when participants chose the Altruistic Benefactor in A3S1 and A1S1 conditions using the Mann‐Whitney U Test, to examine whether and how the Relative Self‐payoff changed as the efficiency of the Altruistic Benefactor increased.

### FMRI Data Acquisition and Preprocessing

Images were acquired using a 3T Prisma Siemens scanner (Siemens AG, Erlangen, Germany) with a 64‐channel head coil at Peking University (Beijing, China). T2‐weighted echoplanar images (EPI) were obtained with blood oxygenation level‐dependent (BOLD) contrast. Sixty‐two transverse slices of 2.3 mm thickness that covered the whole brain were acquired using a multiband EPI sequence in an interleaved order (repetition time = 2000 ms, echo time = 30 ms, field of view = 224 × 224 mm^2^, flip angle = 90°). FMRI data preprocessing was conducted using Statistical Parametric Mapping software SPM12 (Wellcome Trust Department of Cognitive Neurology, London). Images were slice‐time corrected, motion corrected, resampled to 3 mm × 3 mm × 3 mm isotropic voxels, and normalized to MNI space using the EPInorm approach in which functional images were aligned to an EPI template, which was then nonlinearly warped to stereotactic space. Images were then spatially smoothed with an 8 mm FWHM Gaussian filter, and temporally filtered using a high‐pass filter with a cutoff frequency of 1/128 Hz.

### Whole‐Brain Cross‐Study Multivariate Neural Expressions: Verifying the Modulation Effect of Efficiency on the Trade‐Off Between Communal and Obligation Feelings at Neural Level—General Linear Model 1 (GLM1)

A general linear model (GLM) analysis was conducted at the individual level (i.e., first‐level analysis) in SPM12. A design matrix was built with separable run‐specific partitions. In GLM1, the Choice period was focused on and modeled the brain responses of this period in the three Efficiency conditions as three separate regressors, starting from the time the benefactor's information and reciprocal efficiency were revealed and spanning the time that the participant made the reciprocal partner choice: 1) A1S3, i.e., higher reciprocal efficiency of the Strategic Benefactor, where the efficiency of Altruistic Benefactor was 1, while that of Strategic Benefactor was 3, 2) A1S1, i.e., equal reciprocal efficiency, where both benefactors had an efficiency of 1, and 3) A3S1, i.e., higher reciprocal efficiency of the Altruistic Benefactor, where the efficiency of Altruistic Benefactor was 3, while that of Strategic Benefactor was 1. Regressors of no interest included Outcome Period (onset of the Outcome Period showing the two Benefactors’ Costs, 3‐5 s), Allocation (onset of the Allocation Period, starting from the time the rating screen presented and spanning to the time that the participant finished allocation), Miss_Choice (the missing decision period for reciprocal partner choice), and Miss_Allocation (the missing decision period for allocation). Six movement parameters were included as regressors of no interest. All regressors were convolved with a double gamma hemodynamic response function (HRF), and high‐pass temporal filtering was applied with a default cutoff value of 128 s to eliminate low‐frequency drifts. Three contrasts were defined corresponding to the simple effects of the three efficiency conditions.

### Whole‐Brain Cross‐Study Multivariate Neural Expressions: Verifying the Modulation Effect of Efficiency on the Trade‐Off Between Communal and Obligation Feelings at Neural Level—Cross‐Study Neural Expressions

At the behavioral level, the modulation effect of efficiency was observed on the trade‐off between communal and obligation feelings behind reciprocal partner choices. Further, this finding was verified by conducting cross‐study neural expressions for the processing of communal and obligation feelings using the whole‐brain multivariate pattern maps established by the recent research.^[^ ^47^ ^]^ This previous study adopted a perspective of partner control. As stared above, the interpersonal task of this study was similar as the current study, except that participants were paired with only one anonymous benefactor (co‐player) with either altruistic or strategic intention in each round, and decided how much to reciprocate to this benefactor from self‐endowments. Based on paradigm, this previous study develops computational models to predict the amount of reciprocity by quantifying the tradeoff between the latent motivations of self‐interest, communal concern (consisting of guilt & gratitude), and obligation. Then, using MVPA with principal components regression with five‐fold cross‐validation,^[^ ^174^, ^175^, ^176^ ^]^ this previous study trained two separate whole‐brain multivariate pattern maps predictive of communal concern and obligation terms in the behavioral computational model. Three important points regarding the relationship between the present study and the recent research should be clarified. First, the present study and the recent research share a similar experimental foundation, i.e., both involving the generation and weighting of communal and obligation emotions after receiving help, which was the foundation for conducting cross‐study neural expressions of the two emotional motivations. Second, despite the involvements of similar emotional motivations, the present study and the recent research differ fundamentally in the type of reciprocal choice participants make based on these emotional motivations. Consequently, cross‐study neural expressions could contribute to addressing the novel scientific questions proposed in the current study. Third, the two studies constitute entirely separate datasets. The fact that neural representations of communal concern and obligation could be generalized across independent samples provides robust, converging evidence for the reliability of the findings.

Cross‐study neural expressions were carried out in Python 3.6.8 using the NLTools package version 0.4.7 (https://nltools.org/). Specifically, for each participant, the dot‐product of the contrast map was computed for each Efficiency condition (A1S3, A1S1, and A3S1) with the whole‐brain multivariate pattern map for the processing of communal concern established in the recent study.^[^ ^47^ ^]^ These pattern expression values reflected the neural predicted processing of communal concern in the corresponding Efficiency condition. Similarly, for each participant, the dot‐product of the contrast map was computed for each Efficiency condition (A1S3, A1S1, and A3S1) with the whole‐brain multivariate pattern map for the processing of obligation established in the recent study.^[^ ^47^ ^]^ These pattern expression values reflected the neural predicted processing of obligation in the corresponding Efficiency condition. This whole‐brain based multivariate neural expression analysis had been effectively applied in previous research on social emotions to predict both the types and intensity of cognitive activities reflected in neural responses,^[^ ^60^, ^66^ ^]^ and was considered to yield better predictive performance compared to region‐of‐interest (ROI) based neural expression approaches.^[^ ^177^, ^178^ ^]^

These pattern expression values of communal concern and obligation were Min‐Max normalized and entered into the computation of “the neural relative weight of Communal Concern”, i.e., ‘|theneuralpredictedprocessingofcommunalconcern|(|theneuralpredictedprocessingofcommunalconcern|+|theneuralpredictedprocessingofobligation|)’, which reflected the neural trade‐off between communal and obligation feelings when participants made reciprocal partner choices.

To examine the robustness of the main effect of Efficiency and the linear increase effect from A1S3 to A1S1 and A3S1 on the neural relative weight of Communal Concern, permutation analyses were conducted in addition to ANOVA.^[^ ^179^, ^180^ ^]^ Specifically, for each time of the 10000 permutations, condition labels were randomly permuted within each participant, and the ANOVA statistics and linear contrast coefficients were recalculated for each iteration to generate an empirical null distribution. The empirical *p*‐value was defined as the proportion of permuted statistics exceeding the observed value (observed *F* = 3.355 for the main effect; observed *β* = 0.012 for the linear contrast). This approach allows direct estimation of the likelihood that the observed effects could arise by chance. The main effect of Efficiency remained significant in the permutation analysis (permutation *p* < 0.001; Figure S5, Supporting Information). Importantly, the linear trend showing a monotonic increase from A1S3 to A3S1 was also robust (permutation *p* = 0.007; Figure S5, Supporting Information). These results confirmed the robustness of these findings.

### ROI Based Intra‐Subject Representational Similarity Analysis: Identifying the Neural Bases Underlying the Influence of Reciprocal Efficiency—General Linear Model 2 (GLM2)

GLM2 was built and applied intra‐subject representational similarity analysis to identify the neural bases underlying the influence of efficiency on reciprocal partner choices. In GLM2, the Choice period was focused on and divided the brain responses of this period into 27 separate regressors of 3 (Efficiency: A1S3, A1S1, A3S1) × 9 (Benefactor's Cost: 4, 6, 8, 10, 12, 14, 16, 18, and 20) conditions, starting from the time the benefactor's information and reciprocal efficiency were revealed and spanning the time that the participant made the reciprocal partner choice (R1‐R27). Regressors of no interest were the same as GLM1. All regressors were convolved with a double gamma hemodynamic response function (HRF), and high‐pass temporal filtering was applied with a default cutoff value of 128 s to eliminate low‐frequency drifts. The contrast maps corresponding to the 27 regressors of interest were extracted for each participant and used for subsequent analyses.

### ROI Based Intra‐Subject Representational Similarity Analysis: Identifying the Neural Bases Underlying the Influence of Reciprocal Efficiency—Representational Similarity Analysis (RSA)

RSAs were conducted in Python 3.6.8 using the NLTools package version 0.4.7 (https://nltools.org/). To search for the specific brain regions that were involved in the effects of efficiency on reciprocal partner choices, an a priori 200‐parcel whole‐brain parcellation was used based on meta‐analytically functional co‐activation of the Neurosynth database^[^ ^26^, ^60^, ^110^, ^181^ ^]^ (parcellation available at http://neurovault.org/images/39711/) and divided each contrast image for each condition and each participant into 200 parcels. The parcellation scheme, in contrast to the traditional searchlight method, provides a number of advantages, including lower computational demands and better alignment with the functional neuroanatomy.^[^ ^26^, ^181^, ^182^ ^]^ Importantly, it had demonstrated efficacy in multivariate analyses of studies on the neural mechanisms of social emotions and reciprocity.^[^ ^26^, ^60^, ^181^ ^]^ Next, a dissimilarity matrix was created for neural activations in each parcel (the Parcel Dissimilarity Matrix) using pairwise correlation dissimilarity between each pair of the 27 conditions. The Parcel Dissimilarity Matrix represented the neural patterns of each participant across different conditions, serving as indices of neural representations.

**Figure 5:**
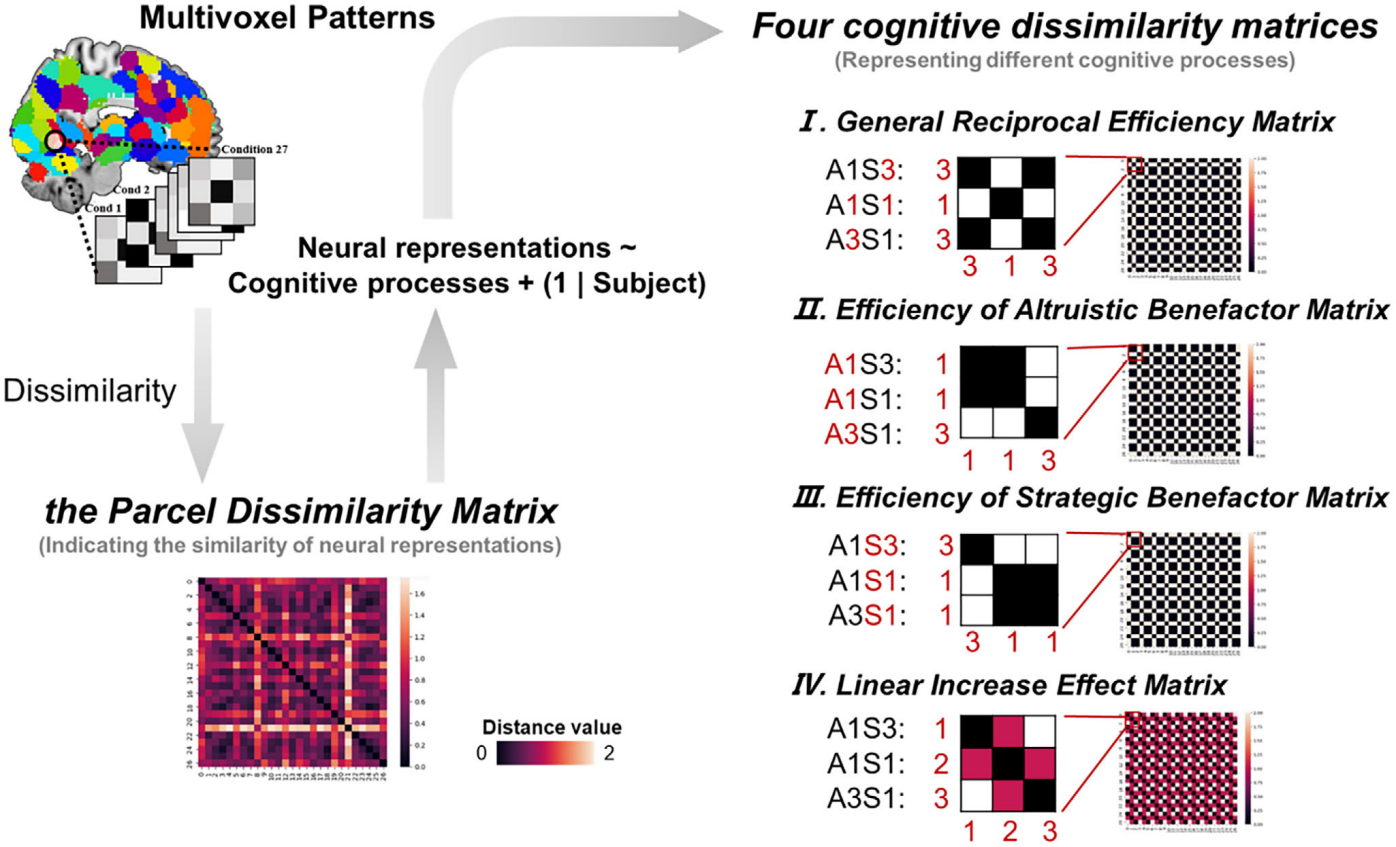
Representational Similarity Analysis. We used an a priori 200‐parcel whole‐brain parcellation based on meta‐analytically functional co‐activation of the Neurosynth database^[^ ^26^, ^60^, ^110^, ^181^ ^]^ and divided each contrast image for each condition and each participant into 200 parcels. For each parcel, we constructed a *Parcel Dissimilarity Matrix* using pairwise correlation dissimilarities across the 27 experimental conditions, representing neural pattern dissimilarities and thus serving as neural representational indices. To assess the correspondence between neural and cognitive representations, we created *four cognitive dissimilarity matrices*, reflecting different psychological processes related to reciprocal efficiency: (I) General Reciprocal Efficiency Matrix, coding A1S3 and A3S1 as ‘3’ (high efficiency), and A1S1 as “1” (low efficiency); (II) Efficiency of Altruistic Benefactor Matrix, coding A1S3, A1S1, and A3S1 as 1, 1, and 3, respectively; (III) Efficiency of Strategic Benefactor Matrix, coded as 3, 1, and 1 for A1S3, A1S1, and A3S1, respectively; (IV) Linear Increase Matrix, coded as 1, 2, and 3 for A1S3, A1S1, and A3S1, respectively. This matrix captures a linear trend across three reciprocal conditions, consistent with observed patterns in reciprocal partner choice and the relative weight of Communal Concern. Then, we transformed the lower triangles of all matrices into vectors and conducted linear mixed‐effects modeling,^[^ ^183^ ^]^ regressing neural dissimilarity vectors on each cognitive matrix separately, with random intercepts for participants. Beta weights and p‐values were extracted from each parcel, and statistical significance was assessed using the Benjamini–Hochberg FDR correction (*p* < 0.05, two‐tailed). Given prior evidence linking efficiency processing to striatal activity,^[^ ^98^ ^]^ we also conducted ROI‐based analyses (FDR‐corrected, *p* < 0.05) in four striatal subregions: the putamen, caudate, and pallidum (defined by the AAL atlas)^[^ ^184^ ^]^ using the WFU PickAtlas tool,^[^ ^185^ ^]^ and the ventral striatum (defined via the Neurosynth database).

Also, four cognitive dissimilarity matrices (**Figure** **5**) were generated to capture different psychological processes related to reciprocal efficiency, serving as indices of cognitive representations to characterize different cognitive processes: 1) General Reciprocal Efficiency Matrix. This matrix reflected the general processing of reciprocal efficiency, which was independent of the type of the benefactor. Therefore, it was designed to identify regions where activity was dissimilar in the low‐efficiency condition (A1S1) compared to the two high‐efficiency conditions (A1S3 and A3S1), but similar between the two high‐efficiency conditions. The A1S3 condition and A3S1 condition were labeled as “high efficiency” conditions, denoted as “3”, while the A1S1 condition was labeled as “low efficiency” condition, denoted as “1”; 2) Efficiency of Altruistic Benefactor Matrix. This matrix characterized the processing associated with the efficiency of the Altruistic Benefactor. The neural activity of brain regions identified should be dissimilar in the A3S1 condition compared to A1S1 or A1S3, but similar between A1S1 and A1S3. In this matrix, the three conditions, “A1S3, A1S1, and A3S1”, were coded as ‘1, 1, and 3′, respectively; 3) Efficiency of Strategic Benefactor Matrix. This matrix characterized the processing related to the efficiency of Strategic Benefactor. The neural activity of brain regions identified should be dissimilar in the A1S3 condition compared to both A1S1 and A3S1, but similar between A1S1 and A3S1. In this matrix, the three conditions, “A1S3, A1S1, and A3S1”, were coded as ‘3, 1, and 1′, respectively; 4) Linear Increase Effect Matrix. In this matrix, the three conditions, “A1S3, A1S1, and A3S1”, were coded as ‘1, 2, and 3′ respectively, which corresponded to the pattern of linear increase across the A1S3, A1S1, and A3S1 conditions that observed in the results for reciprocal partner choices and the relative weight of Communal Concern. This matrix captured the processing related to the effect of efficiency on reciprocal partner choices (Figure 2A) and the underlying trade‐off between the two emotional motives (Figure 2B). It was critical to note that all RDMs were based on the pairwise dissimilarity between conditions; thus, the absolute values of the codes were irrelevant, and only the pattern of similarities and dissimilarities they create matters. The different neural correlates identified by the three RDMs were thus a direct and expected consequence of their unique and dissociable representational structures.

For each of the 200 parcels, linear mixed models^[^ ^183^ ^]^ were utilized to estimate the correlations between the Parcel Dissimilarity Matrix (representing the neural representations) and the four cognitive dissimilarity matrices (representing the cognitive processes) separately (Figure 5). Specifically, the lower triangles of all matrices were transformed into vectors and a regression analysis was performed where the response vectors (i.e., neural activations within each parcel) were regressed on the predictor vectors (i.e., cognitive dissimilarity matrices), with random intercept of each participant included. To assess the statistical significance across the group, the beta weights and *p*‐values for each parcel were extracted, and then conducted Benjamini‐Hochberg False Discovery Rate (FDR) Correction (*p* < 0.05, two‐tailed). Moreover, previous research^[^ ^98^ ^]^ had identified the association between efficiency and neural activities in the striatum. Therefore, regions of interest (ROIs) based analysis (FDR correction, *p* < 0.05) was conducted using four sub‐regions of the striatum, including the putamen, caudate, and pallidum from AAL templates,^[^ ^184^ ^]^ using the WFU PickAtlas Tool^[^ ^185^ ^]^ and the ventral striatum from the Neurosynth database (http://neurovault.org/images/39711/).

### Statistical Analysis

All behavioral and neuroimaging statistical tests were two‐tailed with a significance threshold of α = 0.05, except for the permutation tests, which were one‐tailed as they estimate the probability of observing an effect equal to or greater than the observed statistic. The Greenhouse–Geisser correction was applied when the sphericity assumption was violated in repeated‐measures ANOVAs. All ANOVA analyses were conducted in SPSS 23.0, linear mixed‐effects models (LMMs) were implemented in R, and neuroimaging data analyses were performed using Python 3.6.8 and the NLTools (version 0.4.7) package unless explicitly stated.

## Conflict of Interest

The authors declare no conflict of interest.

## Author Contributions

R.L. and X.Li. contributed equally to this work. R.L., X.Li, X.Z., and X.G. designed the experiments; R.L. and X.Li implemented the study and collected data; R.L., X.Li, X.Liu, Y.N., and X.G. performed analyses; R.L., X.Li, X.Liu, Y.N., X.Z., and X.G. wrote the manuscript; X.Z. and X.G. supervised the project. All authors provided critical revisions and approved the final version.

## Supporting information



Supporting Information

## Data Availability

Original behavioral data from all studies and the unthresholded first‐level maps from the fMRI study are available on GitHub (https://github.com/Regina‐lr/Reciprocal_Efficiency). Raw imaging data are available from the corresponding authors upon request due to privacy concern.
