# Supplementary material for: Efficiency Recalibrates Social‐Emotional Trade‐Offs Behind Partner Choice in Direct Reciprocity through Intention‐Specific Neural Bases
Source: Adv Sci (Weinh). 2025 Nov 9;13(5):e16509. doi: 10.1002/advs.202516509 (PMC12849932; doi:10.1002/advs.202516509)
Supplement: Supplementary file 1 — Supporting Information [file ADVS-13-e16509-s001.docx]

Supplementary Materials for

**Efficiency Recalibrates Social-Emotional Trade-offs behind Partner Choice in Direct Reciprocity through Intention-Specific Neural Bases**

Rui Liao ^1^†, Xintong Li ^2^†, Xuqi Liu ^1^, Yu Nan ^1^, Xiaolin Zhou ^1^, Xiaoxue Gao ^1^*

*Correspondence to:

Xiaoxue Gao (gxx114455@gmail.com or xxgao@psy.ecnu.edu.cn)

**The PDF file includes:**

**Supplementary Methods**

Experimental procedures of the Additional Behavioral Experiment

**Supplementary Results**

fMRI Experiment

Additional Behavioral Experiment

**Supplementary Figures S1 to S6**

**Supplementary Tables S2 to S3**

Supplementary Methods

***Experimental procedures of the Additional Behavioral Experiment***

To exclude the possibility that reciprocal efficiency modulated the intensities of emotions toward the benefactors, we conducted an additional behavioral experiment (n = 37 participants). The procedure of this behavioral experiment (Figure S4) was identical to the interpersonal task of the fMRI experiment, except that (1) participants were required to evaluate their emotional feelings (i.e., gratitude, guilt, indebtedness, and obligation) and appraisals (i.e., second-order belief and perceived care) after learning the reciprocal efficiency of each benefactor and before deciding whom to reciprocate, and (2) there was no time jittering before or after Choice Period. Besides, this experiment included 2 blocks and the order of the two blocks was counterbalanced between the participants. Each block had 27 trials (54 trials in total), encompassing 9 levels of the Benefactor’s Cost in each of the 3 conditions of Efficiency, respectively. All other trial settings were exactly the same as the interpersonal task of the fMRI experiment.

Behavioral analyses conducted on the additional behavioral experiment were similar to the interpersonal task of the fMRI experiment. Additionally, to investigate whether efficiency directly modulated the intensity of participants’ emotional feelings, we conducted 3 (Efficiency: A1S3, A1S1, A3S1) × 9 (Benefactor’s Cost: 4, 6, 8, 10, 12, 14, 16, 18, and 20) ANOVAs on the subjective ratings obtained from this additional behavioral experiment.

Supplementary Results

***fMRI Experiment***

**Reciprocal efficiency influenced participants’ reciprocal allocation**s

A one-way (Efficiency: A1S3, A1S1, A3S1) repeated-measures ANOVA was conducted on the amounts of allocation to the chosen benefactors (Table S2). Results revealed a significant main effect of Efficiency (*F*_(2, 98)_ = 27.668, *p* < 0.001, *η_p_^2^* = 0.361). Specifically, the reciprocal allocation was significantly higher in A1S1 condition than in A1S3 condition (*mean difference* = 2.275, *Standard Error* = 0.354, *95%CI* = [1.564, 2.986], *p* < 0.001) and A3S1 condition (*mean difference* = 2.486, *Standard Error* = 0.355, *95%CI* = [1.773, 3.199], *p* < 0.001). The difference between A1S3 condition and A3S1 condition was not significant (*mean difference* = 0.211, *Standard Error* = 0.401, *95%CI* = [-0.596, 1.017], *p* = 0.602).

Previous research has revealed that individuals make reciprocity choices by weighing the concern for self-interests against reciprocal motivations (including communal and obligation motivations) (47). It should be noted that the present observation that the amount of reciprocity to the chosen benefactor decreased under A1S3 and A3S1 conditions compared to the A1S1 condition, does not imply that participants were less motivated to reciprocate the benefactor and more care about self-interests as the reciprocal efficiency increased. This is because the analysis here focused on the amount allocated from the participant’s own endowment to the chosen benefactor, which is the value before being multiplied by the reciprocity efficiency. After multiplication by the reciprocity efficiency, the actual monetary amount received by the chosen benefactor was in fact greater under A1S3 and A3S1 conditions than under A1S1 (Table S2; for A1S3 compared to A1S1 condition: *mean difference* = 6.325, *Standard Error* = 0.983, *95%CI* = [4.350, 8.300], *p* < 0.001; for A3S1 compared to A1S1 condition: *mean difference* = 10.452, *Standard Error* = 1.222, *95%CI* = [7.996, 12.908], *p* < 0.001). Given the complexity of psychological motivations reflected in these absolute values of allocation, we argue that the ratio between the remaining payoff for the participant after allocation and the final payoff obtained by the chosen benefactor can serve as a more accurate indicator reflecting changes in the relative weighting of self-interest and reciprocal motivation across conditions. Therefore, we defined the ‘Relative Self-payoff’ as ‘$\frac{\mathrm{the} amount reserved for oneself}{(\mathrm{the} amount reserved for oneself + efficiency * the allocation to the benefactor)}$’, reflecting the relative concern for self-interest in comparison to the concern for the chosen benefactor’s interest, and focused on the corresponding results that have been reported in the main text.

**Absolute contributions of communal and obligation motivations to reciprocal choices for both behavioral and neural analyses**

To explore whether and how reciprocal efficiency modulated the contributions of feelings of communal concern (gratitude and guilt) and obligation (sense of obligation) to reciprocal partner choices, we conducted LMMs and extracted the regression coefficients (*β*s) of communal and obligation factors for predicting the probability of choosing the Altruistic Benefactor in each of the three efficiency conditions respectively. Two one-way repeated-measures ANOVAs (Efficiency: A1S3, A1S1, A3S1) were conducted on the regression coefficients (*β*s) of communal and obligation factors separately (Figure S6A). Consistent with our findings from relative weights (Figure 2B), for the communal factor, results revealed a significant main effect of Efficiency (*F*_(2, 98)_ = 20.476, *p* < 0.001, *η_p_^2^* = 0.295), with a significant linear increase across reciprocal efficiency conditions (from A1S3 to A1S1 and A3S1; *F*_(1,49)_ = 22.171, *p < 0.001*, *η_p_^2^* = 0.312). For the obligation factor, the main effect of Efficiency was not significant (*F*_(2, 98)_ = 2.235, *p* = 0.112, *η_p_^2^* = 0.044), although the linear increase trend was marginally significant (from A3S1 to A1S1 and A1S3, *F*_(1,49)_ = 3.268, *p =* 0.077, *η_p_^2^* = 0.063).

Similarly, two one-way repeated-measures ANOVAs (Efficiency: A1S3, A1S1, A3S1) were conducted on the neural expressions of the communal and obligation factors (Figure S6B). For the neural representations of Communal Concern, we observed a significant main effect of Efficiency (*F*_(2, 98)_ = 3.836, *p* = 0.025, *η_p_^2^* = 0.073). For obligation, the main effect of Efficiency was marginally significant (*F*_(2, 98)_ = 2.979, *p* = 0.055, *η_p_^2^* = 0.057). Although the linear trends were not significant (communal factor: *F*_(1,49)_ = 2.273, *p =* 0.138, *η_p_^2^* = 0.044; obligation factor: *F*_(1,49)_ = 1.513, *p =* 0.225, *η_p_^2^* = 0.030), the neural expression of Communal Concern tended to be higher in the A3S1 than in the A1S1 condition (*mean difference* = 0.038, *Standard Error* = 0.015, *95%CI* = [0.008, 0.068], *p* = 0.013), whereas the neural expression of Obligation tended to be higher in the A1S3 than in the A1S1 condition (*mean difference* = 0.036, *Standard Error* = 0.014, *95%CI* = [0.008, 0.065], *p* = 0.014). None of the other simple effects were significant (*ps* > 0.138). These findings are partially consistent with the observations using neural relative weights.

However, previous studies have shown through computational modeling and model comparison that individuals make reciprocal decisions by integrating and weighing the two emotional motives rather than processing them separately (Gao et al., 2024). In this view, relative weights can more effectively capture the extent to which participants tradeoff between these two motivational drivers, thereby offering stronger alignment with the cognitive hypothesis. Therefore, in this article, we mainly focus on the results of relative weights.

***Additional Behavioral Experiment***

**Efficiency influenced participants’ reciprocal choices**

A 3 (Efficiency: A1S3, A1S1, A3S1) × 9 (Benefactor’s Cost: 4, 6, 8, 10, 12, 14, 16, 18, and 20) repeated-measures analysis of variance (ANOVA) revealed significant main effects of Efficiency (*F*_(2, 72)_ = 37.318, *p* < 0.001, *η_p_^2^* = 0.509; Figure S2). Benefactor’s Cost did not show significant main effect on the probability of choosing Altruistic Benefactor (Greenhouse-Geisser correction: *F*_(4.06, 146.15)_ = 1.648, *p* = 0.164, *η_p_^2^* = 0.044), and the interaction effect between these two variables was not significant (Greenhouse-Geisser correction: *F*_(8.31, 299.32)_ = 0.794, *p* = 0.613, *η_p_^2^* = 0.022). Linear trend analyses showed that, the probability of choosing Altruistic Benefactors increased linearly from the A1S3 condition, A1S1 condition to A3S1 condition (*F*_(1,36)_ = 55.616, *p* < 0.001, *η_p_^2^* = 0.607). Similarly, since we did not observe a significant interaction effect between Efficiency and Benefactor’s Cost, and we primarily focused on the effect of Efficiency in the current study, we combined the data of all levels of Benefactor’s Cost in the subsequent analyses.

**Efficiency did not modulate the intensity of participants’ emotional feelings**

Several one-way ANOVAs were conducted to examine the effect of Efficiency (A1S3, A1S1, A3S1) on participants’ subjective ratings, including gratitude, guilt, obligation, indebtedness, perceived care, and second-order belief. Since participants provided separate ratings for the Altruistic Benefactor and the Strategic Benefactor, the data were split accordingly, and analyses were conducted separately for each benefactor type. No significant main effects were observed (Table S1). This suggested us that, efficiency did not directly modulate the intensity of participants’ emotional feelings.

**The relative concern for self-interest did not increase with reciprocal efficiency**

Similarly, to implicitly infer participants’ concerns for self-interest, we also defined the ‘Relative Self-payoff’ as ‘the amount reserved for oneself / (the amount reserved for oneself + efficiency * the allocation to the benefactor)’, representing the relative concern for self-interest in relative to the concern for the chosen benefactor’s interest. For one thing, we compared the Relative Self-payoff when participants chose the Strategic Benefactor in the A1S3 and A1S1 conditions using the Mann-Whitney U Test, which demonstrated how the increased efficiency of the Strategic Benefactor modulated the Relative Self-payoff. Interestingly, results showed that the Relative Self-payoff decreased as the efficiency of reciprocating the Strategic Benefactor increased (*Z* = 1.750, *p* = 0.080; Figure S3). For another, we compared the Relative Self-payoff when participants chose the Altruistic Benefactor in A3S1 and A1S1 conditions, which demonstrated how increased efficiency of Altruistic Benefactor modulated the Relative Self-payoff. No significant difference in the Relative Self-payoff was observed between the A3S1 and A1S1 conditions (*Z* = -1.491, *p* = 0.136; Figure S3).

Supplementary Figures


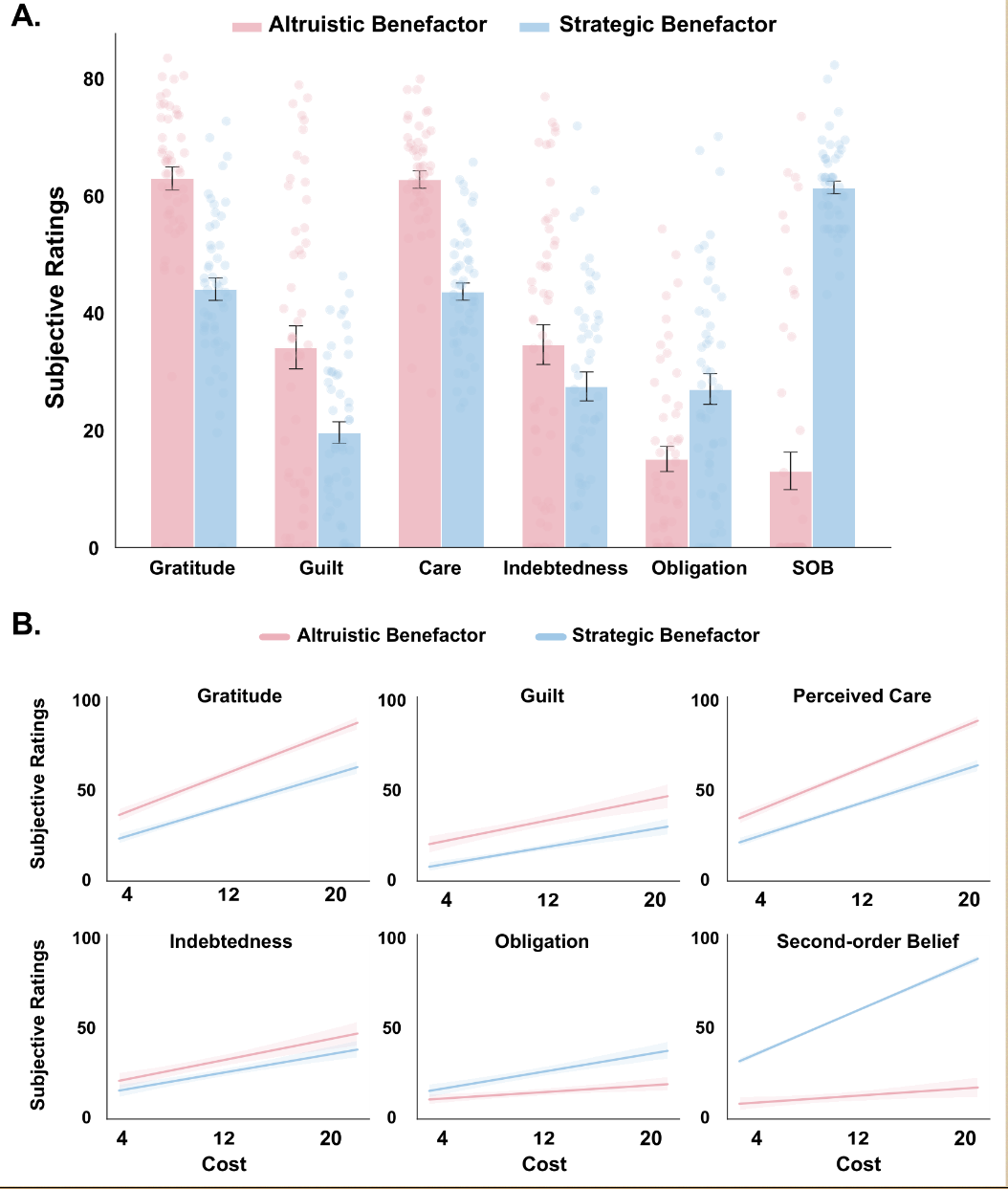


**Figure S1 |** **Subjective Ratings toward the Altruistic Benefactor and the Strategic Benefactor. (A) Participants reported different appraisals (perceived care and second-order belief) and emotions (gratitude, guilt, and obligation) toward the Altruistic and Strategic Benefactors.** Data are presented as mean ± SEM. Each dot represents the average subjective rating for one participant (n = 50). Care refers to perceived care; SOB to second-order belief; Obligation to the sense of obligation. **(B) Subjective ratings for both benefactors increased as the benefactor’s cost increased.** Data (n = 50) are presented as fitted mean values (solid lines) with 95% confidence intervals (shaded areas) generated via nonparametric bootstrapping (1,000 resamples). Pink lines represent the subjective ratings for the Altruistic Benefactor, and the blue lines represents the subjective ratings for the Strategic Benefactor.
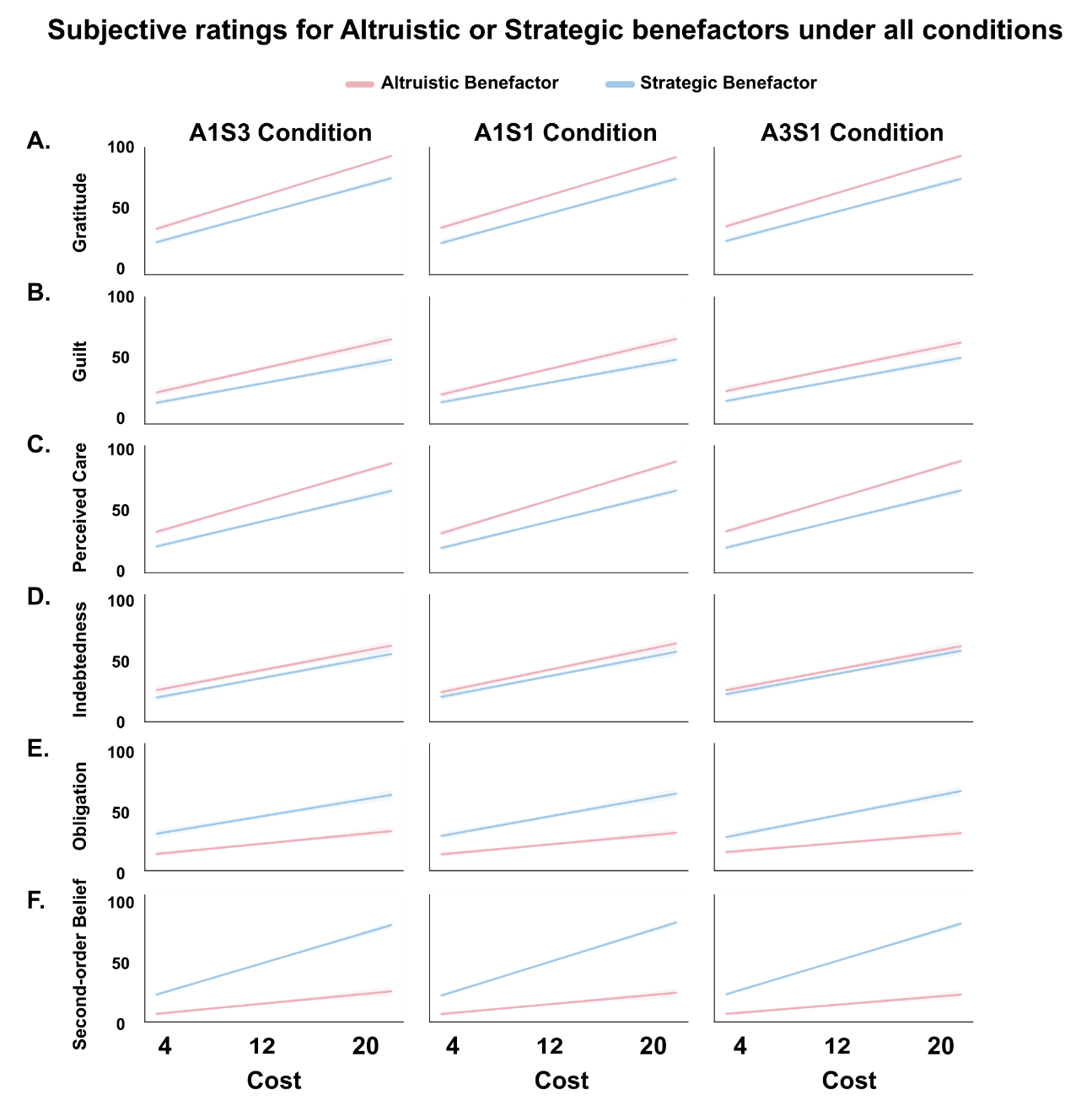


**Figure S2 | Efficiency did not significantly influence participants’ subjective ratings, nor did it show any interaction with Benefactor Type or Benefactor’s Cost.** Regression lines showed that the subjective ratings for both benefactors increased as the benefactor’s cost increased similarly in three reciprocal efficiency conditions. Data of the additional behavioral experiment (n = 37) are presented as fitted mean values (solid lines) with 95% confidence intervals (shaded areas) generated via nonparametric bootstrapping (1,000 resamples). Pink lines represent the subjective ratings for the Altruistic Benefactor, and the blue lines represents the subjective ratings for the Strategic Benefactor.


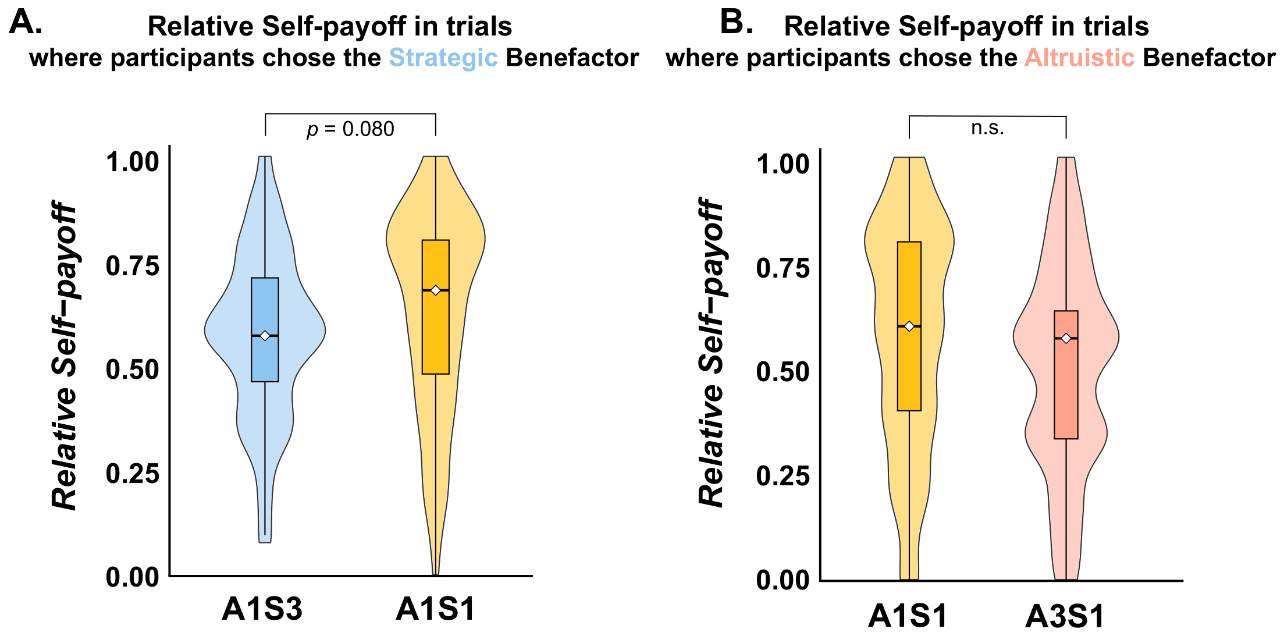


**Figure S3 | The relative concern for self-interest did not increase with reciprocal efficiency. (A)** The Relative Self-payoff did not increase with the higher efficiency of reciprocating to the Strategic Benefactor. **(B)** The Relative Self-payoff did not increase with the higher efficiency of reciprocating to the Altruistic Benefactor. Data of the additional behavioral experiment are shown as violin plots depicting the distribution of individual data points (total n = 37). The white diamond and black bar indicate the mean and the interquartile range, respectively. Statistical comparisons between conditions were performed using two-tailed Mann–Whitney U tests. Data are presented as raw values normalized within participants. *p* values are indicated above the plots. Significance: ‘n.s.’ denotes non-significance (*p* > 0.05).


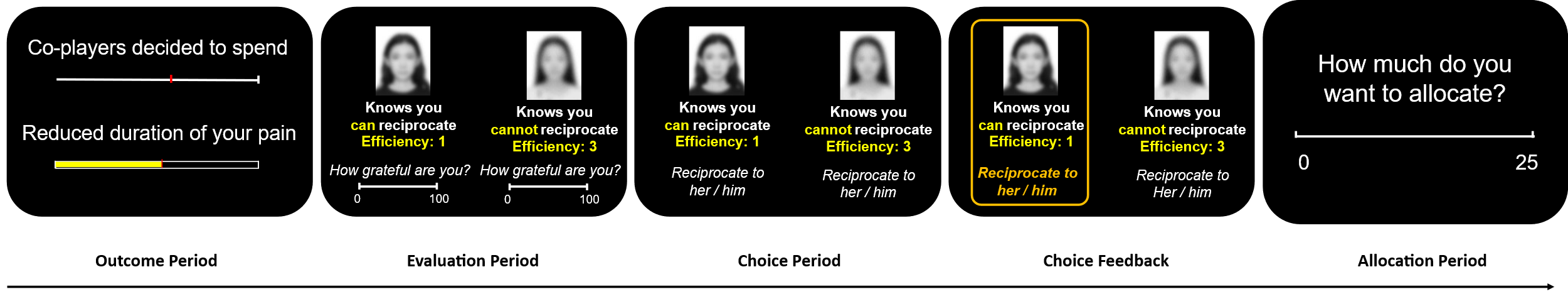


**Figure S4 | Detailed procedure for the additional behavioral experiment.** The procedure of this experiment was identical to the interpersonal task of the fMRI experiment. Similarly, each trial began by informing the participant that the program had randomly chosen two benefactors for the current trial. Then, information regarding the two benefactors’ spending and the corresponding amount of pain reduction for the participant was presented (Outcome Period). Subsequently, participants would see the two benefactors’ blurred photos and IDs, presented on the left and right sides of the screen, respectively. The information that the benefactor knew the participant could or could not repay, as well as the reciprocal efficiency of each benefactor, were given underneath the corresponding photo. Participants were required to evaluate their emotional feelings (i.e., gratitude, guilt, indebtedness, and obligation) and appraisals (i.e., second-order belief and perceived care) toward both benefactors in the Evaluation Period. After that, participant had to choose one benefactor to reciprocate by pressing the left or the right button (Choice Period). A box will appear around the selected benefactor and last for 1 second (Choice Feedback). At the end of each trial, the participant was endowed with 25 yuan and decided how much to allocate to the chosen benefactor as reciprocity (Allocation Period, less than 8 s, continuous choice from 0 to 25, step of 1 yuan). Unbeknownst to the participants, all decisions of the benefactors were predetermined by the computer program.


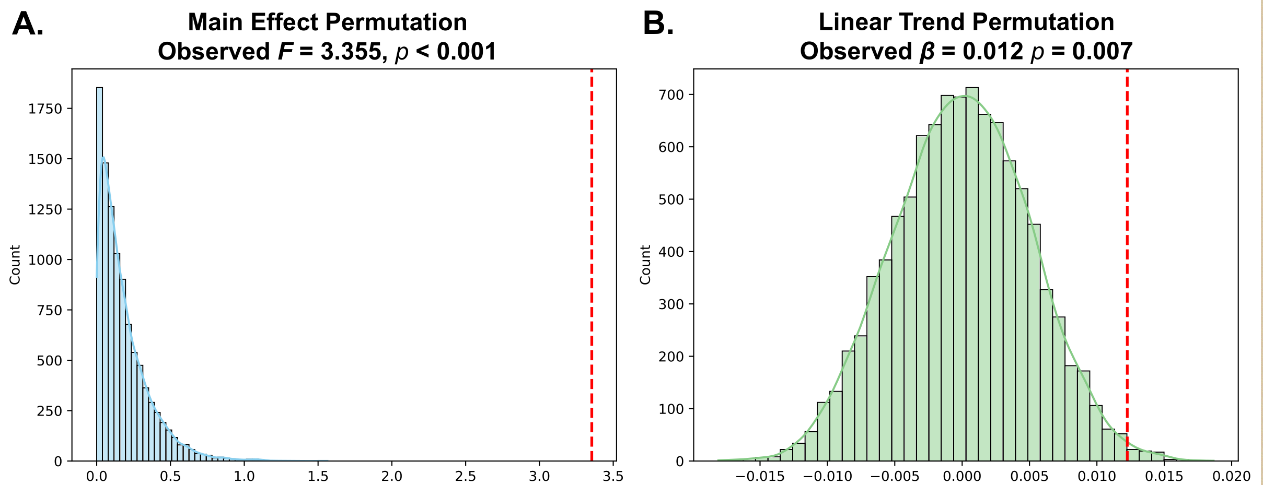


**Figure S5 | Permutation Analysis on the main effect and the linear effect of Efficiency on the neural relative weight of Communal Concern. (A)** The main effect of Efficiency remained significant in the permutation analysis (observed *F* = 3.355, permutation *p* < 0.001). Red dashed line indicates observed F value. **(B)** The linear effect revealed by direct comparison also remained significant in the permutation analysis (observed *β* = 0.012, permutation *p* = 0.007). Red dashed line indicates observed slope. In the permutation analysis, we generated an empirical null distribution by randomly permuting condition labels within each subject (thus preserving the repeated-measures dependency) and recalculating the ANOVA statistics and linear contrast coefficients in each iteration (n = 10,000). The permutation p-values were computed as the proportion of permuted statistics equal to or exceeding the observed statistic in the predicted (one-tailed) direction. This nonparametric approach does not rely on distributional assumptions and directly tests the robustness of the observed effects through data-driven resampling.


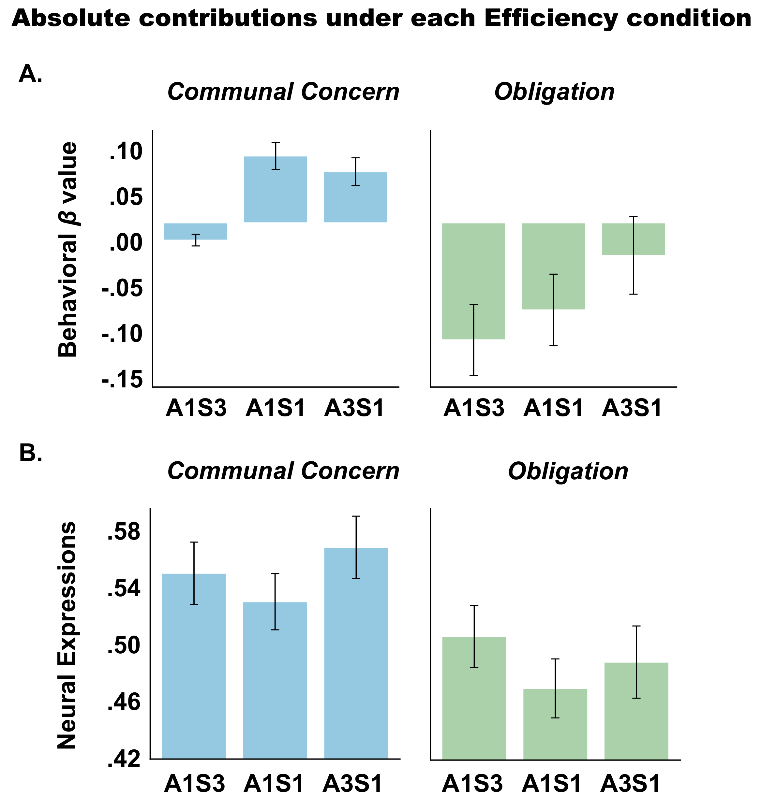


**Figure S6 | Absolute contributions of Communal Concern and Obligation under each Efficiency conditions.** **(A)** Behavioral regression coefficients (β) of Communal Concern and Obligation predicting the probability of choosing the Altruistic Benefactor across three Efficiency conditions (A1S3, A1S1, A3S1). **(B)** Neural expression values of Communal Concern and Obligation across conditions. Data are presented as mean ± SEM, n = 50. Blue bars represent Communal Concern, and green bars represent Obligation. For both behavioral and neural data, one-way repeated-measures ANOVAs (Efficiency: A1S3, A1S1, A3S1) were conducted separately for the two factors, followed by post-hoc pairwise comparisons. All tests were two-tailed with α = 0.05.

Table S1. One-way ANOVAs’ results on participants’ subjective ratings

|  |  | ***F*** | ***(df1,df2)*** | ***Sig*** |
| --- | --- | --- | --- | --- |
| Efficiency | Gratitude | 1.8433 | (2,72) | 0.1657 |
|  | Guilt | 1.0567 | (2,72) | 0.3529 |
|  | Obligation | 0.8199 | (2,72) | 0.4446 |
|  | Indebtedness | 2.4904 | (2,72) | 0.09 |
|  | Second-orderBelief | 0.7022 | (2,72) | 0.4988 |
|  | PerceivedCare | 0.7719 | (2,72) | 0.4659 |
| Efficiency:Cost | Gratitude | 1.3107 | (16,576) | 0.1844 |
|  | Guilt | 1.5913 | (16,576) | 0.0661 |
|  | Obligation | 0.7348 | (16,576) | 0.759 |
|  | Indebtedness | 1.6922 | (16,576) | 0.044 |
|  | Second-orderBelief | 0.7415 | (16,576) | 0.7518 |
|  | PerceivedCare | 1.0723 | (16,576) | 0.3785 |
| Efficiency:Intention | Gratitude | 0.6781 | (2,72) | 0.5108 |
|  | Guilt | 2.0709 | (2,72) | 0.1335 |
|  | Obligation | 0.0841 | (2,72) | 0.9194 |
|  | Indebtedness | 2.2793 | (2,72) | 0.1097 |
|  | Second-orderBelief | 1.7102 | (2,72) | 0.1881 |
|  | PerceivedCare | 3.3007 | (2,72) | 0.0425 |

Table S2. Descriptive statistics of reciprocal choices and allocation.

| **Efficiency Conditions** | **ChoiceA**  **(Mean ± SE)** | **Allocation**  **(Mean ± SE)** | **Benefactor’s Payoff**  **(Mean ± SE)** |
| --- | --- | --- | --- |
| **A1S3** | 0.362 ± 0.053 | 8.709 ± 0.578 | 17.305 ± 1.188 |
| **A1S1** | 0.664 ± 0.051 | 10.971 ± 0.475 | 10.981 ± 0.475 |
| **A3S1** | 0.822 ± 0.043 | 8.497 ± 0.536 | 21.433 ± 1.499 |

Note: ‘ChoiceA’ denotes the probability of choosing Altruistic Benefactor; ‘Allocation’ denotes the monetary allocation that participants reciprocated to the chosen benefactor; ‘Benefactor’s Payoff’ denotes the monetary amount received by the benefactor, which equals the Allocation multiplied by their corresponding reciprocal efficiency.

Table S3. Descriptive statistics of the Relative Self-payoff.

| **Reciprocal Choice** | **Efficiency Conditions** | **Relative Self-payoff**  **(Mean ± SE)** |
| --- | --- | --- |
| **Altruistic** | A3S1 | 0.550 ± 0.026 |
|  | A1S1 | 0.477 ± 0.025 |
| **Strategic** | A1S3 | 0.532 ± 0.025 |
|  | A1S1 | 0.610 ± 0.027 |
